# Integrated Characterization by EDS and Roughness as a Diagnostic Tool for Dental Enamel Degradation: An In Vitro Study

**DOI:** 10.3390/bioengineering13010085

**Published:** 2026-01-12

**Authors:** Cosmin Bogdan Licsăndroiu, Mihaela Jana Țuculină, Petre Costin Mărășescu, Felicia Ileana Mărășescu, Cosmin Mihai Mirițoiu, Raluca Ionela Olaru Gheorghe, Bogdan Dimitriu, Maria Cristina Bezna, Elena Verona Licsăndroiu, Mihaela Stan, Cristian-Marius Bacanu, Ionela Teodora Dascălu

**Affiliations:** 1 Doctoral School, University of Medicine and Pharmacy of Craiova, 200349 Craiova, Romania; licsandroiubogdan@yahoo.com (C.B.L.); ovis45@yahoo.com (R.I.O.G.); cristian.bacanu@umfcv.ro (C.-M.B.); 2 Department of Endodontics, Faculty of Dental Medicine, University of Medicine and Pharmacy of Craiova, 200349 Craiova, Romania; mtuculina@yahoo.com; 3 Department of Dental Prothesis Technology, Faculty of Dental Medicine, University of Medicine and Pharmacy of Craiova, 200349 Craiova, Romania; mihaelastan10@yahoo.com; 4 Department of Orthodontics, Faculty of Dental Medicine, University of Medicine and Pharmacy of Craiova, 200349 Craiova, Romania; ciuca_felicia@yahoo.com (F.I.M.); marceldascalu@yahoo.com (I.T.D.); 5 Department of Applied Mechanics and Civil Constructions, Faculty of Mechanics, University of Craiova, 200512 Craiova, Romania; miritoiucosmin@yahoo.com; 6 Department of Endodontics, Faculty of Dentistry, University of Medicine and Pharmacy Carol Davila Bucharest, 050474 Bucharest, Romania; bogdan.dimitriu@umcfcd.ro; 7 Department of Pathophysiology, Faculty of Dentistry, University of Medicine and Pharmacy of Craiova, 200349 Craiova, Romania; 8 Ciso Medical Bucharest, B-vd Burebista, No. 1, 031106 Bucharest, Romania; licsandroiuverona@gmail.com

**Keywords:** tooth enamel degradation, EDS, roughness, bracket removal, remineralization, diagnostic tool

## Abstract

In fixed orthodontic treatment, brackets are orthodontic attachments bonded to the tooth enamel, and their placement and removal may affect the underlying enamel surface. Enamel degradation is a critical factor for oral health, as it reduces the mechanical strength of teeth and increases susceptibility to caries and erosion. Accurate diagnosis of enamel changes is therefore essential for the evaluation of preventive and restorative treatments. In this study, enamel degradation was investigated via two integrated methods: energy-dispersive X-ray spectroscopy (EDS) and surface roughness measurement. The experimental protocol was performed in three stages: before bracket bonding, after bracket removal, and after applying a remineralization treatment. The experimental design included a repeated-measures structure, with stage (baseline, post-debonding, post-remineralization) as the within-tooth factor and bracket type (sapphire vs. metallic) as the between-tooth factor. Given the violation of the variance homogeneity assumption, group comparisons were ultimately performed using Welch ANOVA followed by Games–Howell post hoc tests, with Bonferroni-adjusted values used for pairwise comparisons. The presence of orthodontic brackets can influence enamel mineralization because the bonding and debonding procedures modify the enamel surface microtopography. These procedures can generate microcracks and surface irregularities, which may affect mineral exchange between enamel and the surrounding environment. In our study, bracket removal led to a significant decrease in the mean atomic percentages of Ca (from 32.65% to 16.37% for sapphire) and P (from 16.35% to 8.60% for sapphire), accompanied by a sharp increase in surface roughness. After remineralization, Ca and P levels increased, while roughness decreased. However, neither the mineral content nor the surface topography fully returned to the initial values, indicating that remineralization achieved only a partial recovery of enamel integrity. These findings highlight that the integrated EDS approach and roughness analysis offer a promising descriptive framework for assessing enamel degradation and monitoring the effectiveness of remineralization therapies. The generated mathematical model provides a powerful descriptive framework for the in vitro data obtained, correlating roughness with mineral composition and treatment stage. However, such a high goodness-of-fit (R^2^ > 0.98) should be interpreted cautiously due to the risk of overfitting. Therefore, rigorous external validation is mandatory before this model can be considered a reliable predictive tool. It also highlights the importance of enamel remineralization therapies after orthodontic treatment, but also the importance of choosing personalized treatment strategies adapted to the enamel type.

## 1. Introduction

Fixed orthodontic treatment commonly uses brackets, which are attachments bonded to the enamel surface to engage the archwire and move teeth. Mechanical, chemical, and microbial factors in the oral cavity are major contributors to the development of simple and complex carious lesions. Tooth enamel serves as the primary protective barrier against these challenges. Due to its high degree of mineralization, tooth enamel is considered to be the hardest and most resistant tissue in the human body. In its composition, tooth enamel contains about 92–96% inorganic substances, mainly in the form of hydroxyapatite crystals that are arranged in prismatic structures. Tooth enamel contains 3% water, the remaining 1% being organic matter involved in the processes of amelogenesis (formation and maturation of enamel). Therefore, from a functional point of view, tooth enamel provides the protection of the crown of the teeth, thus contributing to maintaining the morphological and implicitly functional integrity of the tooth. Due to the fact that ameloblasts disappear after teeth erupt in the oral cavity, tooth enamel can no longer regenerate [1,2,3,4,5,6].

In fixed orthodontic treatment, brackets are bonded to the enamel to hold the archwire and transmit forces to the teeth. After bracket removal (debonding), residual adhesive is eliminated to restore the enamel surface appearance and texture as closely as possible to its original state [7]. However, the literature has argued that the process of mechanical removal of the residual composite after debonding can have a significant and permanent negative impact on the morphological integrity of the tooth enamel. In vitro and in vivo studies have reported that this process can cause microcracks and irregularities on the enamel surface affecting not only dental esthetics but also oral health over a long period of time. The increased roughness of the enamel surface caused by the removal of the residual adhesive after debonding is an important factor contributing to the demineralization of enamel. Thus, the accumulation and retention of plaque is facilitated, resulting in the final occurrence of carious lesions after the completion of orthodontic treatment [8,9,10,11].

Demineralization of vestibular surfaces of enamel, which occurs after removal of the residual adhesive that results after debonding, is a frequent and persistent problem in orthodontic practice. This structural alteration of enamel leads to the appearance of chalky white spots on its surface [12,13,14,15]. During orthodontic treatment, the incidence of new carious lesions was reported to exceed 45% and the total prevalence of carious lesions in these patients is estimated to be over 68%. These data highlight a high enamel vulnerability that occurs during fixed orthodontic treatments and emphasize the importance of implementing rigorous prophylactic measures that help maintain enamel mineralization and prevent the occurrence of incipient carious lesions [16].

The procedure for the complete removal of adhesive residues after debonding takes time, and improper handling of the instrumentation can lead to the appearance of microcracks or enamel injuries [17,18]. However, the degree of damage to tooth enamel after removal of the adhesive can be reduced by using appropriate techniques. The selection of the instrumentation used for the removal of adhesive residues should be carried out according to the hardness and thickness of the adhesive residue, as well as the morphological and structural peculiarities of the tooth enamel. The choice of the method of removing adhesive residues, whether rotary (use of tungsten carbide mills, fine diamond cutters, or abrasive disks) or minimally invasive (laser, air abrasion), must be individually adapted to make the removal of the material more efficient without compromising the integrity of the enamel [9,19,20].

The objective of this in vitro study is to experimentally evaluate the process of tooth enamel degradation after debonding and remineralization by using two complementary methods of investigation: energy-dispersive X-ray spectroscopy (EDS) and surface roughness measurement.

We analyzed three experimental stages. In Stage 1, we determined the Ca and P content of the tooth enamel and measured enamel surface roughness before bracket bonding. In Stage 2, the same measurements were repeated after one month of fixed orthodontic treatment immediately after bracket removal. In Stage 3, we assessed the same parameters after completion of the remineralization protocol to evaluate whether enamel properties could be restored towards their baseline values (before bracket bonding). For a comparative evaluation, we used sapphire brackets and metal brackets in the study.

## 2. Materials and Methods

### 2.1. Selection and Ethical Approval

A total of 200 intact permanent teeth (incisors, canines, premolars, and molars) were used in the in vitro experimental study. The teeth were extracted for orthodontic and periodontal reasons and were collected from patients who were treated at the Oral and Maxillofacial Surgery Clinic of the County Emergency Clinical Hospital Craiova, Romania. The procedures were carried out according to the principles of research ethics, with the approval of the Ethics Commission (Nr.152/11.07.2022) of U.M.F Craiova, in accordance with the principles of the Helsinki Declaration, and in compliance with the rules for the use of human biological material, obtaining the informed consent of patients for the use of extracted teeth for scientific purposes.

The criteria for inclusion in the study:

Intact permanent teeth were used in the study.

Exclusion criteria for the study:

- deciduous teeth;
- fractured permanent teeth;
- teeth with carious lesions or pronounced occlusal wear.

### 2.2. Experimental Design and Workflow

**Figure 1:**
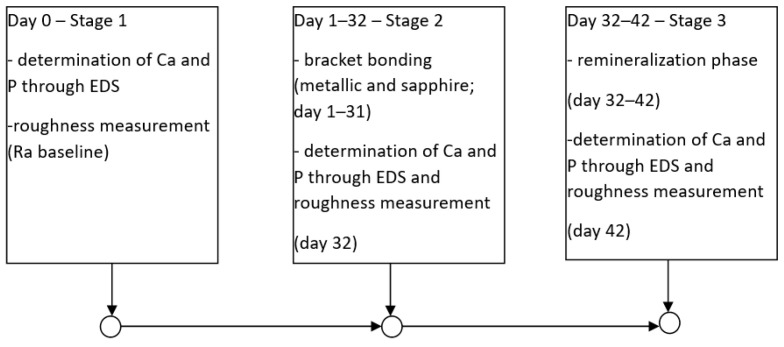
Experimental timeline and evaluation stages. Day 0—Stage 1: Baseline determination of Ca and P through EDS and initial roughness (Ra) measurement. Day 1–32—Stage 2: Period including bracket bonding (Day 1–31) and the post-debonding evaluation (Day 32) immediately following bracket removal and clean-up. Day 32–42—Stage 3: Period including the remineralization phase (Day 32–42), with final determination of Ca and P and roughness measurement performed on Day 42.

In this study, we defined three evaluation stages (Stage 1–Stage 3) to assess enamel degradation by determining calcium and phosphorus levels using energy-dispersive X-ray spectroscopy (EDS) and by measuring enamel surface roughness (Figure 1).

The experimental workflow followed the timeline illustrated in Figure 1, comprising three distinct evaluation stages. Stage 1 was conducted on Day 0 to establish baseline measurements for Ca and P content and surface roughness (Ra). Stage 2 covers the active treatment period from Day 1 to Day 32, during which bracket bonding was maintained, with the actual debonding and subsequent measurements performed on Day 32. Stage 3 encompasses the 10-day remineralization phase from Day 32 to Day 42, with the final surface and elemental evaluations recorded on Day 42.

### 2.3. Determination of Sample Size

Prior to conducting the experiment, the required number of teeth for the study was estimated using G*Power software (version 3.1) to ensure an appropriate level of statistical sensitivity [21]. The calculation, designed to compare the three evaluation stages, was based on a one-way fixed-effect ANOVA, considering a moderate effect size (f = 0.30), a type I error rate of 0.05, and a statistical power of 0.80. According to this analysis, a minimum of 22 teeth was required, measured repeatedly across the three stages (i.e., 66 stage-specific measurements in total). In the present study, a total of 200 teeth were included, of which 100 were bonded with metallic brackets and 100 with sapphire brackets. The same teeth were assessed at three consecutive stages: before bracket bonding, after bracket removal, and after remineralization treatment. This design ensured intra-sample comparability and increased the statistical reliability of the results. A post hoc power analysis confirmed that this sample size provided a statistical power of 1.0, indicating that the study was more than adequately powered to detect significant differences.

### 2.4. Preparation and Disinfection of Teeth

After the extraction, the teeth were washed with distilled water to remove residual debris from their surface. This stage was followed by disinfection by immersion in hydrogen peroxide (H_2_O_2_) 10% for 10 min. This decontamination method was selected on the basis of available literature data confirming the efficiency of the procedure and the minimal effect on enamel morphology at the specified concentration and duration [22]. Although prolonged exposure or use of higher concentrations may induce enamel surface changes, under the experimental conditions applied in this study, the risk was considered clinically insignificant. After the disinfection stage, the teeth were kept in deionized water at a temperature of about 23 °C for a day to prevent dehydration of the enamel.

### 2.5. Allocation of Experimental Groups

Before the bracket bonding stage, the teeth were extracted from the deionised water, carefully dried, and randomly assigned to two equal experimental groups (n = 100 each):

Group A: Teeth on which sapphire brackets were bonded (n = 100).

Group B: Teeth on which metallic brackets were bonded (n = 100).

All samples were handled under standardized laboratory conditions in order to avoid errors in enamel surface analysis.

It should be noted that the total sample of 200 teeth included a heterogeneous collection of types of permanent teeth (incisors, canines, premolars, and molars) extracted for various clinical reasons (orthodontic and periodontal). Although this approach increases biological variability and reflects a pragmatic clinical situation, it is a potential confounding factor given the inherent differences in enamel thickness and coronary morphology. A rigorous random allocation was applied in order to statistically distribute this variability in a balanced way between Group A (sapphire) and Group B (metallic). Therefore, the main objective of the study was to establish a general correlation model: Ra (average roughness) vs. Ca/P and to evaluate average trends rather than accurately quantify the damage on a particular tooth type.

### 2.6. Bracket Bonding and Photopolymerization

Both experimental groups followed the same standard bracket bonding protocol. Transbond™ XT etching gel (3M, Saint Paul, MN, USA) was applied to the vestibular enamel surfaces for 30 s to achieve acid etching, then thoroughly rinsed with water for 10 s and gently air-dried. A thin and homogeneous layer of Transbond™ XT Light Cure adhesive (3M, MN, USA) was then applied and light-cured for 10 s using a Demi Plus LED lamp (Kerr Dental, Kloten, Switzerland).

The brackets were positioned on the vestibular enamel surfaces with 3M™ Transbond™ XT adhesive (Minnesota Mining and Manufacturing Company, MN, USA) and additionally light-cured for 10 s on each side to ensure complete polymerization of the material [21,22]. To reduce experimental variability, a uniform and controlled pressure was applied when bonding all brackets, and all steps of the bonding protocol were kept identical for all samples.

### 2.7. Debonding Procedure and Post-Treatment Analysis

Then, the brackets were maintained on the teeth for 31 days, and on Day 32, they were removed using a special plier (Hu-Friedy, Chicago, IL, USA; ORTHO PLIERS, Long Handle, code 678-220). The procedure was performed by applying an average compressive force of approximately 50 N, in accordance with current clinical protocols [21,22,23].

#### Cleaning of Residual Glue

Immediately after debonding (removal of brackets with pliers), the residual composite adhesive was completely removed from the enamel surface. The speed of the counter-angle part at which the milling is mounted is an essential factor in the removal of the residual composite. Although low-speed rotary tools cause vibrations and discomfort to patients, studies in the literature have shown that they achieve more effective removal of the adhesive and the occurrence of lower enamel roughness compared to high-speed rotary tools [24,25,26].

For this reason, in our study, the removal of the adhesive was carried out using a tungsten conical carbide cutter with 12 blades (H375R-21-016, Galaxy 12 Fluted Long Taper, 8 mm, OrthoTechnology, West Columbia, SC, USA) mounted at a low-speed counter-angle piece (16,000 rpm), under constant water cooling, until the visual removal of the composite [27].

**Figure 2:**
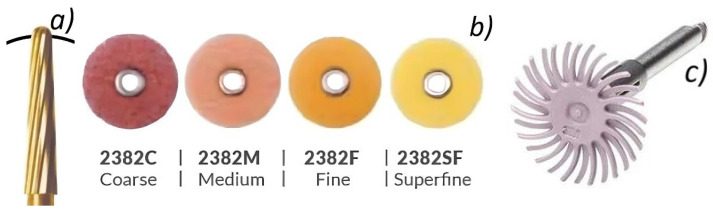
Instruments used for adhesive cleaning and finishing: (**a**) tapered 12-blade bur; (**b**) coarse, medium, fine, and superfine Sof-Lex disks; (**c**) pink Sof-Lex spiral wheel (https://www.orthotechnology.com).

Sugsompian et al. [28] reported that Sof-Lex disk polishing generated smooth and even enamel surfaces with only minor damage to the surface. For this reason, in our study, after removing the residual adhesive, at the polishing stage, we opted to use Sof-Lex disks of medium, fine, and superfine grain (3M™ ESPE, MN, USA). During this stage, we applied light-to-moderate pressure for 15–20 s. For medium grain size, abrasive disks were used at an average speed of 10,000 rpm. For fine and superfine granulation, the average speed used was 15,000–20,000 rpm. During the course of this polishing step, we maintained a unidirectional and continuous movement. Thus, the appearance of grooves at the level of the enamel surface was prevented. For the final polishing, we used pink Sof-Lex spiral wheels (3M™ ESPE, MN, USA). They were fitted to a low-speed counter-angle piece (15,000–20,000 rpm). (Figure 2). This speed range has proven effective for achieving similar shine to that obtained by using polishing paste, eliminating the need for its use [27,28].

### 2.8. Protocol of Remineralization

After completion of adhesive removal from the enamel surface and the corresponding assessments at Stage 2 (after bracket removal), enamel remineralization was initiated. On the vestibular enamel surface of each tooth, a thin and uniform layer of GC Tooth Mousse^®^ remineralizing gel (GC Dental, Luzern, Switzerland; GC Corporation, Tokyo, Japan; 900 ppm fluoride) was applied with a sterile microbrush. The gel was left in place for 3 min and then removed by gentle rinsing with distilled water, according to the manufacturer’s instructions. This application was performed once daily for 10 consecutive days to reproduce a short-term clinical protocol recommended by the manufacturer. During the 10-day treatment, all samples were stored in artificial saliva (0.4 g/L NaCl, 0.4 g/L KCl, 0.795 g/L CaCl_2_·2H_2_O, 0.78 g/L NaH_2_PO_4_·2H_2_O, 1 g/L urea, pH 7). This medium was selected to provide stable, ion-rich conditions favorable to the remineralization process and to more closely reproduce the physiological conditions in the oral cavity than storage in deionized water. Artificial saliva was maintained at 37 °C and renewed daily to ensure constant ion availability.

### 2.9. Instrumentation

For the investigation of enamel surface degradation, the following analysis tools were used:

1. Preliminary analysis of tooth enamel surfaces was performed using an OLYMPUS optical microscope (Olympus Corporation, Tokyo, Japan) equipped with STREAM ESSENTIALS image analysis software (version V2.1).

2. For enamel surface imaging (morphological assessment), a Phenom Pure ProX scanning electron microscope (Thermo Fisher Scientific, Waltham, MA, USA) was used. Elemental analysis (Ca and P) was performed by energy-dispersive X-ray spectroscopy (EDS) using the detector integrated in the SEM (Element Identification software, version 2.0). The device operated at an acceleration voltage of 15 keV, and micrographs were obtained at magnifications of ×1000 and ×2000. The 3D Roughness Reconstruction module (version 2.0) was used to analyze the SEM images and to calculate surface roughness parameters.
3. The same Phenom Pure ProX scanning electron microscope, equipped with a backscattered-electron detector and the 3D Roughness Reconstruction module (version 2.0), was used for qualitative and morphological analyses of the enamel surface. The device operated at an acceleration voltage of 15 keV, and the micrographs were obtained at magnifications of ×1000 and ×2000.

### 2.10. Correlation Between the Contents of Ca and P and the Surface Roughness of Tooth Enamel

In this study, EDS measurements were performed directly on the treated enamel surfaces, without additional polishing, in order to preserve the clinically relevant surface morphology created by bracket bonding, debonding, and remineralization. Because the enamel surfaces were not flattened and polished, the EDS data should be considered semi-quantitative. Consequently, Ca and P contents and the Ca/P ratio were used mainly for relative comparisons between the three stages (before bracket bonding, after bracket removal, and after remineralization treatment), rather than for absolute elemental quantification. In order to integrate both methods (EDS and roughness analysis) as a diagnostic tool for tooth enamel degradation, we attempted to establish a correlation function between enamel surface roughness (Ra) and the semi-quantitative Ca and P contents obtained by EDS using regression analysis. Regression analysis is a statistical approach used to evaluate the association between a dependent variable (also called the response variable—surface roughness in this study) and one or more independent variables (also known as predictors or explanatory variables—percentages of Ca and P in this study).

### 2.11. Choosing and Preparing the Surface for Microscopic Examination

First, the enamel surface of the teeth was examined at all three experimental stages (before bracket bonding, after bracket removal, and after the remineralization process) using an optical microscope.

**Figure 3:**
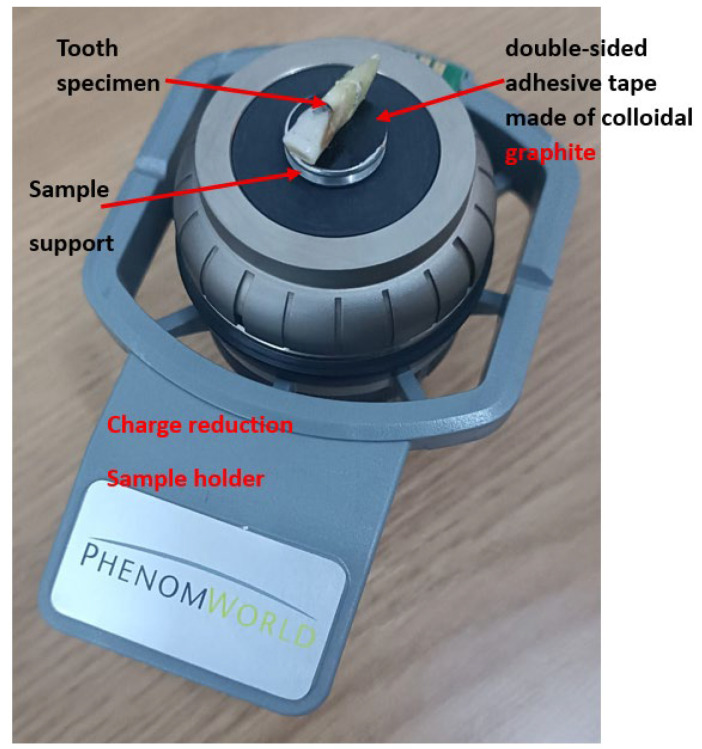
Load-dissipation support device, which includes the dental sample and its mounting base.

The purpose of this part of the study was to characterize the morphological changes in the tooth surface at each experimental stage. Enamel surface morphology was then examined in greater detail using a scanning electron microscope (SEM), while Ca and P contents were assessed by energy-dispersive X-ray spectroscopy (EDS) attached to the SEM. Finally, tooth enamel surface morphology was quantified by measuring surface roughness using the Ra parameter. SEM examinations were carried out on the vestibular enamel surfaces of intact teeth, focusing on the areas where orthodontic brackets had previously been bonded. For each experimental group, the area showing the most pronounced surface irregularities after bracket removal was selected for detailed imaging. Before examination, the teeth were fixed to aluminum supports using double-sided conductive carbon tape to ensure firm grip and optimal electrical conductivity. The prepared specimens were subsequently positioned in a load-dissipation holder, as illustrated in Figure 3, and inserted into the SEM chamber for analysis. This method allows the examination of isolated samples without the need for metal (e.g., gold) or carbon coating, thus preserving the natural topography of the surface.

To ensure the reproducibility of measurements across all three stages, the ROI was initially centered on the vestibular surface where the bracket was to be bonded. After debonding (Stage 2) and remineralization (Stage 3), the area was repositioned using microscopic landmarks and residual markings from the bracket base, which were subsequently cleaned. The scanning area for both EDS and 3D reconstruction was strictly limited to this ROI to guarantee that the detected changes in mineral content and topography reflect the exact site of orthodontic intervention.

### 2.12. Microscope Imaging, EDS Analysis, and 3D Roughness Reconstruction

At each of the three experimental stages (Stage 1: before bracket bonding, Stage 2: after bracket removal, and Stage 3: after remineralization treatment), enamel surface morphology was first assessed by optical microscopy. Images at 100× magnification were acquired with an OLYMPUS optical microscope (Olympus Corporation, Tokyo, Japan) using STREAM ESSENTIALS software (version 2.1) and saved in JPEG format.

Subsequently, the same regions of interest on the vestibular enamel surface were examined with a Phenom Pure ProX scanning electron microscope (Thermo Fisher Scientific, Eindhoven, The Netherlands) equipped with an energy-dispersive X-ray spectroscopy (EDS) detector. SEM/EDS was performed on the native enamel surface at each stage, without mechanical flattening or polishing, to avoid removing the superficial layer affected by bracket debonding and/or by the remineralization treatment. To minimize charging-related artifacts on this non-conductive substrate, imaging and EDS acquisition were carried out using the instrument’s charge-reduction conditions recommended by the manufacturer, and identical acquisition settings were maintained across all samples and stages [29,30]. Because measurements were acquired on an unpolished, non-planar surface, EDS results were treated as semi-quantitative and interpreted for relative comparisons across stages under identical acquisition conditions rather than as absolute compositional values.

The EDS atomic percentages of Ca and P were obtained using the software’s built-in quantification and were aggregated per tooth as the mean of the measurement points within a predefined region of interest (ROI). This ROI (measuring approximately 4 × 4 mm) was consistently located at the center of the vestibular enamel, corresponding to the bracket bonding site. By maintaining the same ROI across Stage 1, Stage 2, and Stage 3, we ensured that the relative comparisons of Ca and P accurately represent the local mineralization dynamics.

High-resolution SEM micrographs of the enamel surface were processed using the 3D Roughness Reconstruction module of Phenom ProSuite software (version 2.0). This module applies a shape-from-shading algorithm to generate a three-dimensional reconstruction of the analyzed area and to calculate surface roughness parameters with submicrometric accuracy from backscattered-electron images. In this study, we used Ra (arithmetical mean roughness) and Rz (mean peak-to-valley height) calculated along selected linear profiles within the reconstructed area to describe changes in enamel surface morphology between the three stages. Because the enamel surfaces were examined in their native, unpolished condition, significant surface height variations were present, especially after bracket debonding. Consequently, SEM images may show regions with different apparent focus within the same field of view, reflecting real surface roughness rather than imaging artifacts. To minimize surface charging effects, imaging was conducted under low-vacuum conditions and at a reduced accelerating voltage [31]. These parameters allowed stable image acquisition without the need for conductive coating, ensuring that both surface topography and elemental composition could be evaluated on the same areas [31].

### 2.13. EDS Analysis

The EDS analysis was carried out according to ASTM E1508–12(2018) standard specifications [32].

In order to ensure a representative analysis and to prevent selection bias, the analysis points have been established according to a random sampling method. For this purpose, from a predefined grid (4 × 4 matrix) that was superimposed over the area of interest of each sample, a set of potential positions was superimposed over the ROI (region of interest) of each sample. From the 16 possible grid intersections, 14 analysis points were selected through controlled random selection to ensure a uniform and representative distribution of the elemental composition across the entire examined area.

The average of these points was used to calculate the overall composition of the surface. The 14-point measurements showed high repeatability and comparable compositional profiles within each condition. Quantitative results (Ca, P, and Ca/P) are therefore reported as averaged atomic percentages derived from these 14 points. In addition, for illustrative purposes, one representative EDS spectrum acquired from a single chosen point is shown for each experimental condition (qualitative reference only). The chosen spectrum accurately reflects the relative proportions of the chemical elements, providing an overview of the chemical composition of the sample and facilitating further interpretation of the results. The EDS analysis was designed as a comparative assessment, meaning that identical acquisition parameters and analytical conditions were applied to all samples and experimental stages, allowing relative comparison of elemental changes rather than absolute stoichiometric determination.

### 2.14. Analysis of Enamel Surface Roughness

The enamel surface texture assessment was carried out using the parameters Ra (arithmetical mean roughness) and Rz (mean peak-to-valley height) in accordance with standard SR EN ISO 21920-2:2022 [33]. Although both indicators were determined, the Ra parameter was chosen for further statistical analysis due to its broad recognition and frequent use in the literature on dental materials. This parameter is considered as a reliable index for the characterization of general surface roughness, thus allowing relevant comparison of the results with those reported in previous research [34].

### 2.15. Statistical Analysis

All datasets were compiled in Microsoft Excel and analyzed in GraphPad Prism (v10.2.0; GraphPad Software, San Diego, CA, USA). Each tooth was assessed at three consecutive stages (Stage 1: baseline, Stage 2: post-debonding, Stage 3: post-remineralization); therefore, the data were analyzed as repeated measurements. The experimental design included one between-subject factor, bracket type (sapphire vs. metallic; n = 100 teeth per type), and one within-subject factor, stage (three levels).

For each outcome variable (Ca, P, Ca/P, Ra, and Rz), a two-way repeated-measures ANOVA was performed. When the sphericity assumption was violated, the Geisser–Greenhouse correction was applied.

The primary analysis for differences between stages and bracket types was designed as a two-way repeated-measures ANOVA (or mixed-effects model if sphericity was violated). However, due to the critical non-homogeneity of variances detected by the Levene test (*p* < 0.01), the primary comparison between the three stages (A1–A3, B1–B3) was conducted using the robust Welch ANOVA test, followed by the Games–Howell post hoc test, as these methods do not assume equality of variances. This approach allowed for reliable detection of significant differences despite the increased variability post-debonding. For direct comparisons between bracket types at the same stage (e.g., A2 vs. B2), one-way ANOVA with Bonferroni-adjusted post hoc comparisons was used [35].

When repeated-measures ANOVA assumptions were not met, the data were analyzed using a mixed-effects model (REML) with tooth as a random effect. Normality was evaluated using the Kolmogorov–Smirnov test and homogeneity of variances using the Brown–Forsythe test. Prespecified post hoc comparisons were performed (i) between stages within each bracket type and (ii) between bracket types at the same stage, with Bonferroni correction for multiple testing. Bonferroni-adjusted values were considered statistically significant. However, due to non-homogeneity of variances detected by the Levene test (*p* < 0.01), further comparisons between groups were made using the Welch ANOVA test, followed by the Games–Howell post hoc test for pairwise comparisons, both of which do not assume equality of variances. A materiality threshold was uniformly adopted for all primary analyses (Welch ANOVA and Games–Howell tests). For all post hoc pairwise comparisons involving multiple simultaneous tests, the Bonferroni correction was applied (adjusted α = 0.0033), and only adjusted *p* values below α = 0.0033 were considered statistically significant.

Group A1: includes experimental results for 100 teeth before bonding sapphire brackets;

Group A2: includes experimental results for 100 teeth after removal of sapphire brackets;

Group A3: includes experimental results for 100 teeth after removal of sapphire brackets and application of remineralization treatment;

Group B1: includes experimental results for 100 teeth before bonding metallic brackets;

Group B2: includes experimental results for 100 teeth after removing metal brackets;

Group B3: includes experimental results for 100 teeth after removing metal brackets and applying remineralization treatment.

In cases involving multiple comparisons, Bonferroni correction was used to adjust the level of significance, thus reducing the likelihood of false positives. The adjusted alpha value was calculated by dividing the value of 0.05 by the total number of paired tests performed.

## 3. Results

The results presented in the following sections reflect the average response of the heterogeneous samples described in the methodology. Due to the mixed composition of the sample (including incisors, canines, premolars, and molars), data on composition (EDS) and roughness (Ra) represent general statistical trends, assuming that the random allocation has distributed the inherent structural variability of teeth between groups in a balanced manner.

### 3.1. Analysis of Tooth Enamel Surface Morphology with Scanning Electron Microscope and EDS

**Figure 4:**
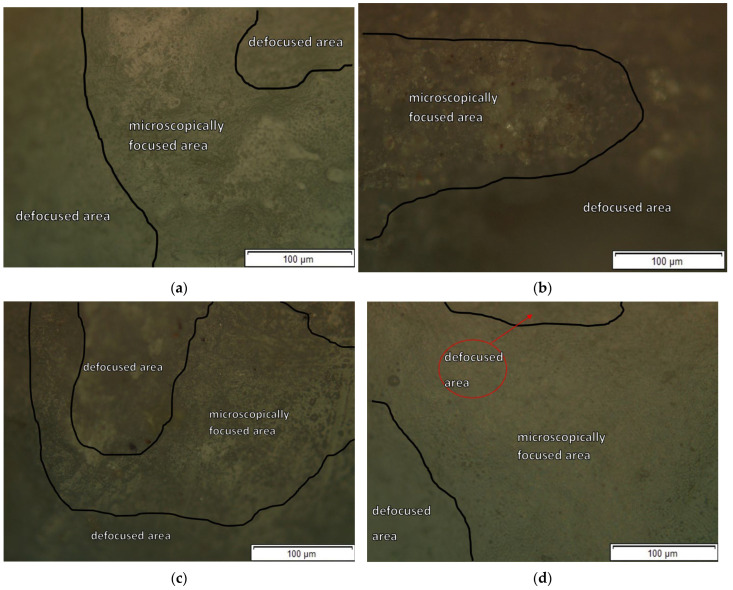

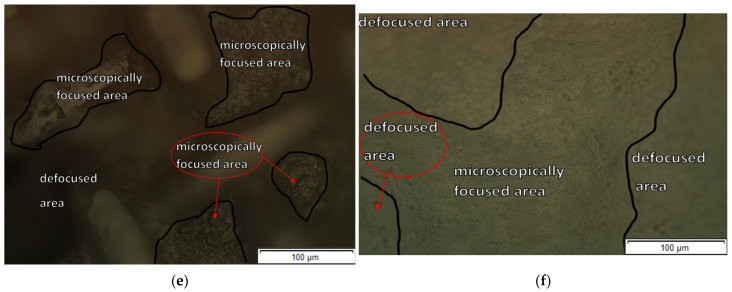
Optical microscopy images illustrating enamel morphology in focused and defocused regions (focused = in focus within focal plane) where the brackets were bonded: (**a**) Enamel surface before bonding the sapphire bracket; (**b**) Enamel surface after debonding the sapphire bracket; (**c**) Enamel surface after the remineralization process; (**d**) Enamel surface before bonding the metallic bracket; (**e**) Enamel surface after debonding the metallic bracket; (**f**) Enamel surface after the remineralization process.

In the first stage, the analysis of enamel morphology was carried out using optical microscopy and scanning electron microscopy. Subsequently, to illustrate the surface changes at the microscopic level, representative teeth (one tooth with a metal bracket and another with a sapphire bracket) were analyzed in detail in each group. Representational images for each of the three analysis stages are shown in Figure 4a–c for a sapphire bracket tooth, and in Figure 4d–f for a metal bracket tooth.

From the analysis of the images, significant morphological differences in the enamel surface can be observed between the three stages of examination: before the bracket bonding, after its removal, and after the remineralization treatment. At the initial stage, the microscopically focused area is considerably larger compared to that observed after removal of the bracket. The microscopically focused area refers to the enamel region that can be sharply visualized under the optical microscope, corresponding to the portion of the surface that lies within the focal plane (within the depth of field) at the selected focus setting. In contrast, the defocused area represents regions with higher surface roughness, microcracks, or topographical elevations that fall outside the focal plane of observation, thus appearing blurred in the optical image. The increase in surface irregularities after bracket removal leads to a higher overall roughness compared with the initial enamel state. As a result, the optical microscope can focus only on limited surface zones due to the increased height variation in the enamel microrelief. Following remineralization, a partial reduction in these irregularities was observed, reflected by an enlarged focused area; however, this area remained smaller than in the initial condition. These microscopic findings are consistent with the quantitative roughness measurements, confirming both the enamel surface degradation after debonding and its partial recovery after remineralization.

**Figure 5:**
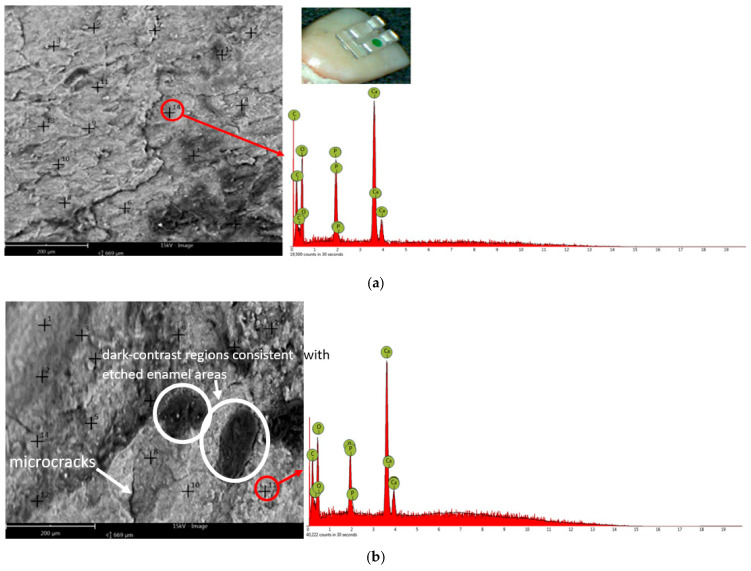

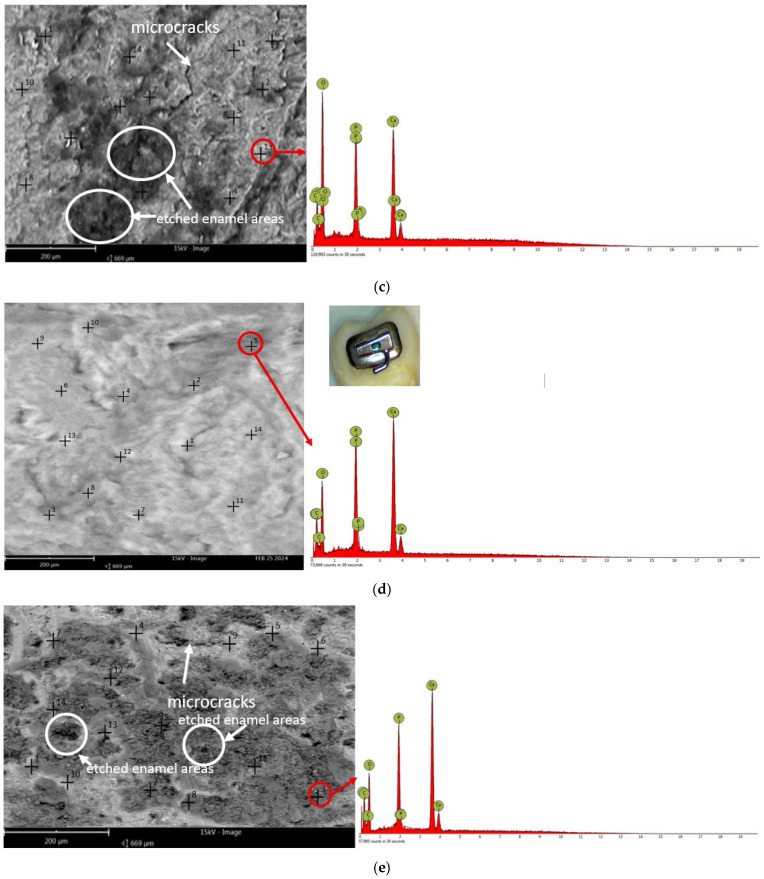

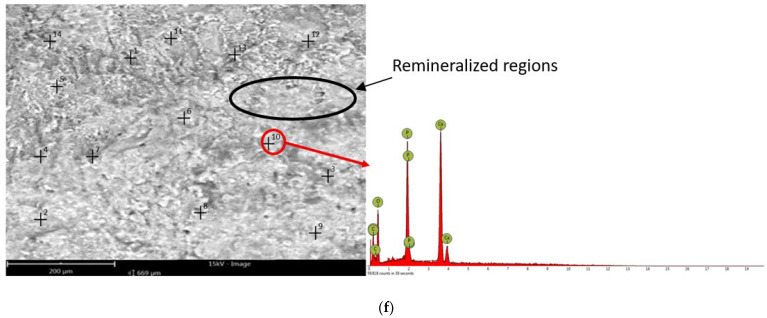
Representative SEM micrographs and corresponding EDS spectra acquired from a selected point on the enamel surface for each experimental condition (two bracket types × three stages). (**a**–**f**) Scale bar = 200 μm. The photographic insert in each panel indicates the macroscopic region of interest on the tooth surface where the microanalysis was performed. Sapphire brackets: (**a**) Stage 1; Inset: macroscopic optical image (×10) illustrating an orthodontic sapphire bracket bonded on the vestibular enamel surface, indicating the region of interest for subsequent SEM/EDS analysis; (**b**) Stage 2; (**c**) Stage 3. Metallic brackets: (**d**) Stage 1; Inset: macroscopic optical image (×10) illustrating an orthodontic metallic bracket bonded on the vestibular enamel surface, indicating the region of interest for subsequent SEM/EDS analysis; (**e**) Stage 2; (**f**) Stage 3.

**Figure 6:**
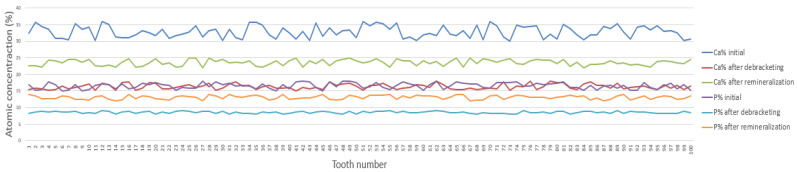
Individual Ca and P atomic percentages (at.%) measured by EDS for teeth with sapphire brackets at Stage 1 (baseline), Stage 2 (post-debonding), and Stage 3 (post-remineralization). Descriptive statistics (mean ± SD) are reported in Table S1; statistical differences are reported in Table 2 and Table 3 (Bonferroni-adjusted *p* values).

**Figure 7:**
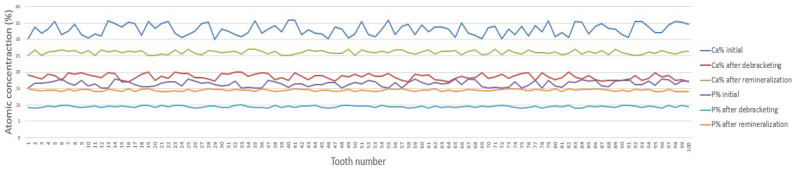
Individual Ca and P atomic percentages (at.%) measured by EDS for teeth with metallic brackets at Stage 1 (baseline), Stage 2 (post-debonding), and Stage 3 (post-remineralization). Descriptive statistics (mean ± SD) are reported in Table S1; statistical differences are reported in Table 2 and Table 3 (Bonferroni-adjusted *p* values).

Analysis of enamel morphology by electron scanning microscopy (SEM) presented in Figure 5 revealed the same trends previously observed by optical microscopy. SEM analysis of the enamel surfaces after bracket removal revealed a higher number of dark regions (darker-contrast areas in the SEM micrographs), consistent with local depressions and microdefects/microcracks and/or residual adhesive traces. These areas indicate localized surface degradation or residual adhesive traces, resulting in a rougher morphology compared with both the initial and post-remineralization stages. After remineralization treatment, these irregularities (highlighted by darker-contrast areas) were partially reduced but not completely eliminated. This result suggests a partial restoration of the enamel structure without a complete return to the original morphology. Figure 5 shows representative SEM micrographs and the corresponding representative EDS spectra acquired from a chosen point on the enamel surface for each experimental condition (two bracket types × three stages). These spectra are provided as qualitative reference. All quantitative comparisons of Ca, P, and Ca/P are based on the averaged atomic percentages from the 14 analysis points per tooth and are presented in Figure 6 and Figure 7. The compositional results obtained by energy dispersion spectroscopy (EDS) are consistent with the morphological observations performed by electron scanning microscopy (SEM). The EDS analysis identified the following major chemical elements: carbon (C), oxygen (O), calcium (Ca), and phosphorus (P). The pronounced decrease in Ca and P content after removal of brackets indicates a mineral loss associated with the enamel demineralization process, resulting in a rough and irregular surface, visible as the darker areas in the SEM images. In this in vitro model, the observed mineral loss is most plausibly related to acid etching during bonding and to the mechanical debonding/adhesive removal procedures, which can alter the superficial enamel layer. In vivo, additional biofilm-related acid challenges may further contribute. After the remineralization treatment, the partial increase in Ca and P concentrations suggests the re-deposition of some minerals, correlated with a partial smoothing of the enamel surface. However, the values remain below those corresponding to the initial stage, indicating that the enamel structure has only been partially restored. For all 14 spectra containing the Ca and P concentrations, the arithmetic mean values were calculated. The centralized mean values of the atomic concentrations of Ca and P for each of the 100 teeth are shown in Figure 6 for sapphire brackets and in Figure 7 for metal bracket teeth.

In Figure 5, the EDS spectra are shown for qualitative reference only; quantitative Ca and P levels are reported in Table 1.

The EDS spectra are shown for qualitative reference only; quantitative Ca, P, and Ca/P results are based on averaged values from 14 analysis points per tooth and are reported in Figure 6 and Figure 7. In Figure 6 and Figure 7, for both types of brackets, sapphire and metal, similar compositional trends can be observed. In all cases, Ca and P levels drop significantly after removal of brackets, confirming a pronounced local demineralization of the enamel surface, followed by a partial recovery after remineralization treatment. These variations are also reflected in the Ca/P ratio, which decreases after bracket removal and decreases further after remineralization, shifting closer to the stoichiometric hydroxyapatite reference (Ca/P ≈ 1.67). However, notable differences were observed between the two types of brackets. Teeth with metal brackets showed higher concentrations of Ca and P at all stages—especially after take-off of brackets and after remineralization—suggesting less aggressive enamel adhesion and less mineral loss compared to sapphire brackets. Instead, sapphire brackets showed more pronounced fluctuations in Ca and P content, reflecting more pronounced surface changes during bracket removal. Overall, the results indicate that metal brackets induce a lower degree of demineralization and allow a more efficient remineralization process than sapphire ones, most likely due to differences between their physicochemical characteristics and enamel adhesion behavior. Previous in vitro studies have reported significantly higher enamel demineralization adjacent to ceramic brackets compared with metal ones [36,37,38,39,40,41,42], which is attributed to differences in surface roughness, adhesive retention, and plaque accumulation at the bracket–enamel interface.

As shown in Table 1, teeth with metallic brackets (Group B) maintained higher mean concentrations of Ca (18.52 ± 0.84) and P (9.42 ± 0.32) after debonding compared to the sapphire group (Group A), suggesting a less aggressive impact on the enamel surface.

### 3.2. Enamel Roughness Findings

As a complementary experimental approach, surface roughness measurements were used to validate observed trends through optical microscopy and SEM/EDS analysis. The results on roughness confirmed the sharp loss of surface uniformity after removal of brackets and partial recovery after remineralization treatment, consistent with variations in Ca and P content evidenced by EDS. To highlight the microscopic changes in the enamel surface, representative teeth selected from each experimental group were examined in detail.

**Figure 8:**
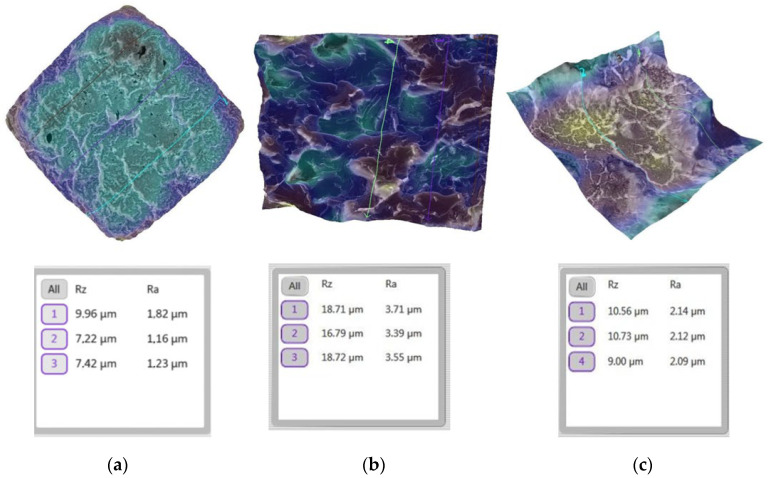
(**a**) Stage 1: 3D surface reconstruction of the enamel in the area where the sapphire bracket was attached, recorded before bonding. The mean Ra value measured within the analyzed zone was 1.4 µm; (**b**) Stage 2: 3D surface reconstruction of the enamel in the area where the sapphire bracket was attached, recorded after removing the bracket. The mean Ra value measured within the analyzed zone was 3.55 µm; (**c**) Stage 3: 3D surface reconstruction of the enamel in the area where the sapphire bracket was attached, recorded after the remineralization treatment. The mean Ra value measured within the analyzed zone was 2.11 µm.

We initially analyzed the surface roughness of the enamel before the bonding of the sapphire bracket (Figure 8a).

The SEM software automatically provides multiple surface roughness parameters during 3D reconstruction of the investigated area. In this study, both Ra and Rz were recorded. Ra is the most commonly used roughness parameter in dental and orthodontic research and was therefore selected for statistical analysis [43], while Rz is a standard engineering descriptor included for descriptive completeness [44]. Ra was used for quantitative statistical analysis, while Rz is reported as a complementary descriptive parameter automatically generated by the SEM software.

A significant increase in surface roughness was recorded after the removal of the sapphire brackets, accompanied by obvious micro-topographic changes and increased values of the parameter Ra (Figure 8b). The remineralization process resulted in a partial smoothing of the enamel surface; however, residual irregularities remained visible (Figure 8c).

A similar analysis was carried out for the teeth on which a metal bracket was bonding.

**Figure 9:**
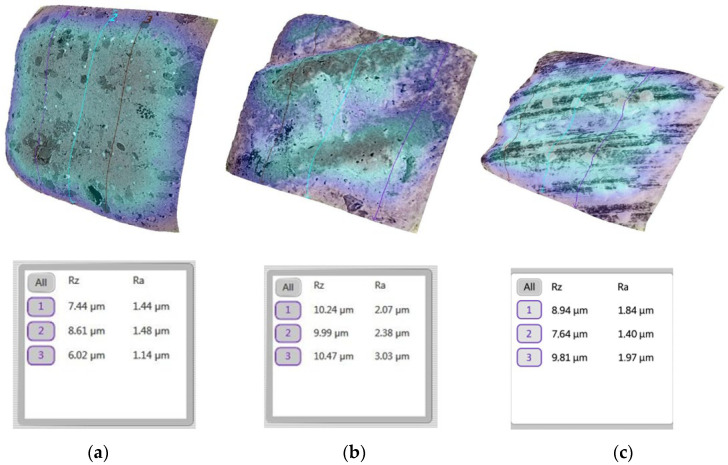
(**a**) Stage 1: 3D surface reconstruction of the enamel in the area where the metallic bracket was attached, recorded before bonding. The mean Ra value measured within the analyzed zone was 1.35 µm; (**b**) Stage 2: 3D surface reconstruction of the enamel in the area where the metallic bracket was attached, recorded after removing the bracket. The mean Ra value measured within the analyzed zone was 2.49 µm; (**c**) Stage 3: 3D surface reconstruction of the enamel in the area where the metallic bracket was attached, recorded after the remineralization treatment. The mean Ra value measured within the analyzed zone was 1.73 µm. Ra denotes the arithmetical mean roughness, while Rz denotes the mean peak-to-valley height; both parameters were defined and interpreted according to SR EN ISO 21920-2:2022.

Figure 9 illustrates the evolution of the surface from a smooth initial state to a rougher texture after the removal of the metallic bracket, and finally to a partially restored state after the remineralization process.

A direct comparison between sapphire and metallic brackets, as illustrated in Figure 8 and Figure 9, shows that sapphire brackets induced higher enamel surface roughness values compared to metallic brackets, particularly at the post-debonding stage. Although both bracket types exhibited a reduction in roughness after remineralization, the Ra values associated with sapphire brackets remained consistently higher, indicating a more pronounced surface alteration of the enamel.

**Figure 10:**
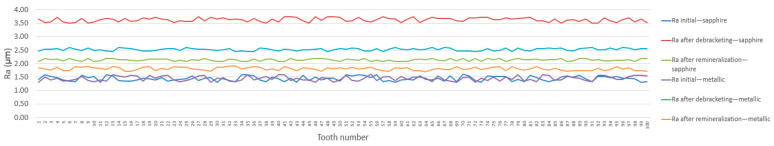
Ra (arithmetical mean roughness, µm) values for all studied teeth on which sapphire and metallic brackets were applied.

All values of the Ra parameter for all studied teeth, on which sapphire and metal brackets were applied, are shown in Figure 10.

The evolution of Ra values (Figure 10) quantitatively validates the morphological trends observed in the 3D reconstructions (Figure 8 and Figure 9). Given the non-homogeneity of variances (Levene test, *p* < 0.01), the robustness of these differences was confirmed through Welch ANOVA (F = 4127.53, *p* < 0.001 for sapphire; F = 5874.22, *p* < 0.001 for metallic), ensuring that the observed increase in roughness after debonding is statistically significant regardless of the high variability in Stage 2.

The significant increase in Ra values after the removal of the brackets confirms the pronounced irregularities of the previously microscopically highlighted surface, as well as the decrease in Ca and P concentrations detected by EDS, indicating local demineralization of enamel. Following the remineralization treatment, the partial decrease in Ra correlates with the smoother morphology observed with SEM and with the partial recovery of Ca and P, suggesting the re-deposition of minerals. Overall, these results demonstrate that the roughness measurements quantitatively validate the microstructural and compositional changes identified by the other experimental methods.

While Figure 8 and Figure 9 provide representative 3D visualizations of specific specimens, Figure 10 illustrates the collective trend across all 200 samples. The direct comparison shows that while both systems achieve partial recovery, the metallic group consistently maintains a smoother topography post-remineralization (1.73 μm) compared to the sapphire group (2.11 μm), a difference confirmed by the Games–Howell post hoc analysis (*p* < 0.001).

### 3.3. Statistical Evaluation and Results of the Experiment

A comprehensive statistical evaluation was carried out to validate the relevance of the detected variations. The initial verification, performed using the Grubbs (0.01) test, indicated the absence of abnormal values in all datasets. Alternately, a post hoc evaluation of statistical power demonstrated that the size of the experimental sample was sufficient to highlight significant differences (power = 1.0). For each group analyzed, the calculated power was equal to 1.0, confirming that the inclusion of 100 teeth per group ensured robust statistical reliability. Full details of the results obtained are given in Table S1 of the Supplementary Materials.

The samples were divided into two main groups according to the bracket material:

• Group A—teeth with sapphire brackets (A1: before bonding, A2: after bracket removal, A3: after remineralization treatment);
• Group B—teeth with metallic brackets (B1: before bonding, B2: after bracket removal, B3: after remineralization treatment).

No statistically significant difference (*p* > 0.05) was observed between the initial roughness of the enamel surfaces of the two groups (A1 vs. B1), thus confirming an equivalent basis of comparison (Figure 6 and Figure 7).

The normality of the data was tested using the Kolmogorov–Smirnov test. All six datasets showed low statistical D values and *p* values higher than 0.01, meaning that the null hypothesis of normality could not be rejected under any circumstances. Therefore, all datasets follow a normal distribution, confirming that the use of parametric statistical tests (such as ANOVA and post hoc t-tests) is statistically justified. The distribution models of Ca and P at all experimental stages (before bracket bonding, after removal, and after remineralization treatment) are compatible with normal distribution, indicating high homogeneity and statistical reliability of the measured data. All data are presented in Table S2 of the Supplementary Materials. The Levene test indicated a lack of homogeneity of variances (*p* < 0.01). Therefore, further comparisons between groups were made using ANOVA Welch and Games–Howell post hoc tests, which do not imply equality of variances. The non-integer degrees of freedom observed in the Welch ANOVA results (such as for the metallic group) arise from the Satterthwaite approximation used by this robust test to correct for the observed non-homogeneity of variances. Crucially, the unusually high values initially reported for the metallic group (Ca and P) were found to be transcription errors and have been corrected to reflect the precise results obtained from the statistical software. This re-verification confirms the highly significant differences between stages, aligning the magnitude of the values with those observed for the sapphire group.

For teeth with sapphire brackets, the following results and interpretations were obtained:

• The Welch ANOVA test indicated a statistically significant difference in calcium concentration between the three experimental stages (F = 1819.6, *p* < 0.001).
• The Games–Howell post hoc analysis showed that all the pair comparisons were significant (*p* < 0.001), demonstrating a steady reduction in calcium content after removal of the bracket, followed by a partial recovery after remineralization (see Table S3 of the Supplementary Materials).
• The Welch ANOVA test indicated statistically significant differences in phosphorus content between the three experimental conditions (F = 2845.7, *p* < 0.001).
• The Games–Howell post hoc tests confirmed that all pair comparisons were significant (*p* < 0.001), showing a sharp decrease in phosphorus content after removal of the bracket, followed by a partial recovery after remineralization (see Table S3 of the Supplementary Materials).

For teeth with metal brackets, the following results and interpretations were obtained:

• The Welch ANOVA test revealed extremely significant differences in calcium concentration between the three experimental stages (F = 3850.55, *p* < 0.001).
• All the pair comparisons made by the Games–Howell post hoc test were also significant (*p* < 0.001), confirming a pronounced reduction of calcium after removal of the bracket, followed by a partial recovery after remineralization (see Table S3 of the Supplementary Materials).
• The Welch ANOVA test revealed extremely significant differences in phosphorus concentration between the three experimental stages (F = 4920.88, *p* < 0.001).
• The Games–Howell post hoc tests confirmed that all pair comparisons were significant (*p* < 0.001), indicating a pronounced reduction in phosphorus after bracket removal and a partial recovery after remineralization (see Table S3 of the Supplementary Materials).

ANOVA unifactorial tests revealed statistically significant differences both in the sapphire bracket groups (A1, A2, A3) (F = 7962.27, *p* < 0.001 for Ca; F = 18,764.51, *p* < 0.001 for P) and in the metal bracket groups (B1, B2, B3) (F = 10,843, *p* < 0.01 for Ca; F = 15,812.39, *p* < 0.001 for P). In addition, direct comparisons showed significant differences between sapphire and metal groups in the stages after removal of brackets (A2 vs. B2: F = 1129.42, *p* < 0.001 for Ca; F = 946.31, *p* < 0.001 for P) and after remineralization (A3 vs. B3: F = 1472.56, *p* < 0.01 for Ca; F = 1018.47, *p* < 0.1 for P).

Post hoc pair comparisons using Bonferroni correction (adjusted = 0.0033) confirmed that almost all differences between stages and bracket types were statistically significant (Table 2 for Ca, Table 3 for P).

**Table 2:** Post hoc pairwise comparisons (Bonferroni correction, adjusted α = 0.0033) showing the mean percentage differences in calcium (Ca) atomic concentration between experimental stages and bracket types (A1–A3: sapphire brackets; B1–B3: metallic brackets).

Comparison	Description	Mean Difference (%)	Statistically Significant (Bonferroni-Adjusted *p* < 0.0033)
A1 vs. A2	Sapphire: Baseline vs. Post-debonding	16.41	Yes
A2 vs. A3	Sapphire: Post-debonding vs. Post-remineralization	−7.23	Yes
A2 vs. B2	Sapphire vs. Metallic (Post-debonding)	−2.52	Yes
A3 vs. B3	Sapphire vs. Metallic (Post-remineralization)	−2.54	Yes
B1 vs. B2	Metallic: Baseline vs. Post-debonding	14.08	Yes
B2 vs. B3	Metallic: Post-debonding vs. Post-remineralization	−7.25	Yes

**Table 3:** Post hoc pairwise comparisons (Bonferroni correction, adjusted α = 0.0033) showing the mean percentage differences in phosphorus (P) atomic concentration between experimental stages and bracket types (A1–A3: sapphire brackets; B1–B3: metallic brackets).

Comparison	Description	Mean Difference (%)	Statistically Significant (Bonferroni-Adjusted *p* < 0.0033)
A1 vs. A2	Sapphire: Baseline vs. Post-debonding	8.01	Yes
A2 vs. A3	Sapphire: Post-debonding vs. Post-remineralization	−4.47	Yes
A2 vs. B2	Sapphire vs. Metallic (Post-debonding)	−0.94	Yes
A3 vs. B3	Sapphire vs. Metallic (Post-remineralization)	−1.44	Yes
B1 vs. B2	Metallic: Baseline vs. Post-debonding	6.89	Yes
B2 vs. B3	Metallic: Post-debonding vs. Post-remineralization	−4.97	Yes

Note: A1—baseline, A2—post-debonding, A3—post-remineralization (sapphire brackets); B1—baseline, B2—post-debonding, B3—post-remineralization (metallic brackets). Statistical significance refers to Bonferroni-adjusted *p* values.

A similar statistical approach was also applied to experimental data obtained from surface roughness measurements. In order to avoid redundancy, statistical calculations based on roughness data were presented in a concise manner. The Grubbs test (α = 0.01) confirmed that no aberrant values were present in any dataset. A post hoc analysis of the statistical power verified that the sample size was adequate to detect significant effects (power = 1.0), indicating that the use of 100 teeth per group provided solid statistical validity.

The Kolmogorov–Smirnov test (α = 0.01) was applied to check whether the data followed a normal distribution. For teeth on which sapphire brackets were bonded, the initial and post-remineralization values showed a normal distribution (*p* > 0.01), while the data obtained after bracket removal did not meet the normality criterion (*p* < 0.01), indicating a greater dispersion of roughness after bracket removal. Consequently, the Box–Cox transformation was applied to this dataset. After applying the Box–Cox transformation (λ ≈ −0.42), the distribution of post-bracket removal values became statistically normal (*p* > 0.01). For teeth with metal brackets, the initial and post-remineralization values showed a normal distribution (*p* > 0.01), while the data obtained after bracket removal did not meet the normality criterion (*p* < 0.01), indicating an increased variability in surface roughness after bracket removal. After applying the Box–Cox transformation to this dataset, values of λ ≈ −0.35 and *p* = 0.184 were obtained, confirming that the distribution became statistically normal. Homogeneity of variances was checked using Levene’s test. The roughness variances for the initial, post-bracket removal, and post-remineralization surfaces were not homogeneous (*p* < 0.01). This result confirms the existence of significant differences in the dispersion of the data, mainly caused by the sharp increase in roughness after bracket removal. Both findings were consistent for teeth with sapphire brackets and those with metal brackets. Subsequently, Welch ANOVA and Games–Howell tests were applied. For teeth with sapphire brackets, the Welch ANOVA test revealed significant differences between at least two conditions (initial, post-bracket removal, and post-remineralization) (F = 4127.532, *p* < 0.001). The Games–Howell post hoc test indicated that enamel roughness increased significantly after removal of the sapphire brackets and partially decreased after remineralization, but did not return to baseline (*p* < 0.001 for all pairwise comparisons). Similar conclusions were obtained for teeth with metal brackets. The Welch ANOVA test confirmed statistically significant differences between the three conditions (F = 5874.219, *p* < 0.001). According to the results of the Games–Howell post hoc test, enamel roughness increased significantly after removal of the metal brackets and subsequently decreased after remineralization, without returning completely to baseline—a pattern similar to that observed with sapphire brackets, but with slightly less variation (*p* < 0.001 for all pairwise comparisons).

In conclusion, the statistical data confirmed the following:

Both types of brackets caused a significant decrease in the atomic concentration of Ca and P. Each type of bracket caused a significant increase in the roughness of the enamel surface. Sapphire brackets produced more pronounced effects (greater mineral loss and higher roughness) compared to metal brackets.

All results confirm that the type of bracket influences the severity of enamel surface changes and changes in mineral composition.

Remineralization was only partially effective, suggesting the need for additional or prolonged treatments to fully restore the enamel structure. Statistical distributions revealed a greater dispersion of the data after bracket removal, indicating an increased variability in the degree of enamel surface damage. Kolmogorov–Smirnov, Levene, Welch ANOVA, and Games–Howell tests indicated statistically significant differences between all conditions analyzed (initial, post-bracket removal, and post-remineralization).

### 3.4. Correlation Between Roughness (Ra), Ca, and P

To simplify the correlation between surface roughness (Ra) and Ca and P concentrations (since establishing a regression function of the form Ra (Ca, P) would be difficult)—the analysis was reduced to a single parameter, namely the determination of a function of the form f(x). In this sense, the Ca/P ratio was chosen, so that the regression analysis aimed to identify a function of the form Ra (Ca/P). Another motivation is that in order to obtain a more relevant interpretation from a chemical and biological point of view, the correlation analysis was moved from the individual Ca and P concentrations to their molar ratio (Ca/P). This ratio represents a fundamental indicator of the mineralization state of dental enamel, since hydroxyapatite—the main mineral component of enamel—has a stoichiometric composition characterized by a Ca/P ratio of approximately 1.67. Deviation from this ideal value reflects ongoing processes of demineralization (lower ratios associated with calcium loss) or remineralization (higher ratios associated with crystalline re-deposition), thus providing more significant information about the structural and compositional balance of enamel than individual elemental values. Consequently, the relationship between surface roughness (Ra) and the Ca/P ratio serves as a more sensitive and direct descriptor of structural changes induced by bracket bonding/debonding processes and remineralization treatments. In addition, the Ca/P ratio is widely used in the literature as a marker of mineral integrity [40]. Because enamel apatite is biologically non-stoichiometric, Ca/P values reported for sound human enamel can deviate from 1.67 [45]; atomic Ca/P ratios in the order of ~1.7–2.1 have been reported, depending on the analyzed region and analytical protocol [38]. Using EDX-based quantification, a Ca/P ratio around 2.13 ± 0.17 has been reported for sound enamel, highlighting that reported values also depend on the quantification mode (atomic% vs. wt%) [42]. The molar ratio of Ca to P in hydroxyapatite is calculated according to Equation (1). However, the “atomic concentration” (atomic %) expresses the relative number of atoms of an element in relation to the total number of atoms, as shown in Equation (2). Analyzing Equations (1) and (2), it can be seen that the atomic ratio is numerically identical to the molar ratio, since both reflect the ratio between the number of atoms of the two elements.

(1) $$\frac{n_{Ca}}{n_{P}}=\frac{molar\;number\;of\;Ca}{molar\;number\;of\;P}$$

(2) Atomic%(i) = (Nᵢ/ΣNⱼ) × 100

where Nᵢ is the atomic concentration (number of atoms) of element i (e.g., Ca or P) obtained from the EDS spectrum, and ΣNⱼ is the sum of the atomic concentrations of all quantified elements in the same spectrum (normalization to 100%).

Descriptive statistics (mean ± SD) for Ca/P are provided in the Supplementary Materials (Tables S1–S4). In the sapphire group, Ca/P decreased from Stage 1 (1.989 ± 0.155) to Stage 2 (1.912 ± 0.102) and Stage 3 (1.806 ± 0.105). In the metallic group, the same trend was observed (Stage 1: 2.008 ± 0.153; Stage 2: 1.963 ± 0.107; Stage 3: 1.800 ± 0.054). Overall, values shift toward the stoichiometric hydroxyapatite reference (Ca/P ≈ 1.67) after remineralization. Initial values between 1.9 and 2.3 reflect the inherent non-stoichiometric nature of biological enamel apatite and inter-sample variability, consistent with the broad range of values reported in the literature for sound human enamel. Stage 1 values above 1.67 are consistent with the non-stoichiometric character of biological enamel apatite and inter-sample variability; therefore, in this study, Ca/P is interpreted primarily in a comparative manner across stages on the same teeth. After remineralization, the ratio visibly approaches the value of 1.67, which means that the process led to the restoration of a mineral composition closer to natural hydroxyapatite. Similar trends can be observed for metal brackets. Initially, the ratio varied between ~1.71 and 2.36, with values often exceeding 1.9 and sometimes even >2.2, indicating a possible initial non-stoichiometric enamel/inter-sample variability. After bracket removal, the ratio ranged between 1.76 and 2.18, showing a slight decrease, but remaining above the ideal value, indicating chemical changes occurring following orthodontic treatment (partial demineralization). This localized demineralization can be attributed to the microstructural damage during bracket bonding and removal, which interfere with normal ionic exchange between enamel and the surrounding medium. After remineralization, the Ca/P ratio in the metallic group ranged from 1.70 to 1.91 (mean ± SD: 1.800 ± 0.054), which is closer to the hydroxyapatite reference value (Ca/P ≈ 1.67) than the corresponding earlier-stage values; however, it remained above 1.67. Both series of samples (sapphire and metal) show the same overall pattern: the Ca/P ratio decreases after bracket removal and shifts closer to the hydroxyapatite reference value (Ca/P ≈ 1.67) after remineralization, although it remains above 1.67. In the metallic group, the post-remineralization Ca/P distribution appears narrower, suggesting a more uniform response across samples under the present experimental conditions. However, this study was not designed to determine the mechanism underlying this difference. A plausible explanation is that the enamel surface condition after bracket removal and adhesive clean-up (e.g., the extent of surface irregularities and the distribution of residual resin) differed between bracket types, which could modulate the effective contact of the remineralizing agent with enamel. Therefore, we interpret this as a descriptive trend that supports reproducibility within our in vitro setup rather than as evidence for a specific physicochemical mechanism.

To determine the function by regression analysis, MathCad software (Parametric Technology Corporation, Boston, MA, USA), version 13.0, was used, in which a database with standard functions was created. Since a direct correlation between surface roughness (Ra) and the Ca/P ratio could not be established with sufficient statistical accuracy (the correlation factor R^2^ being around 0.3), the analysis was refined by introducing a discrete parameter (Ψ) representing the experimental stage (initial Ψ = 1, after bracket removal Ψ = 2, after remineralization Ψ = 3). This approach takes into account the physicochemical processes specific to each stage, allowing the regression model Ra (Ca/P, Ψ) to describe the evolution of surface properties more precisely. The high descriptive power (R^2^ > 0.98) of Equation (3) was achieved by integrating the discrete stage parameter (Ψ), which represents distinct physicochemical states: baseline (Ψ = 1), demineralized (Ψ = 2), and remineralized (Ψ = 3). We acknowledge that such high values in biological data may indicate a risk of overfitting; therefore, this model is presented as a descriptive framework for this specific in vitro dataset, requiring external validation for predictive use.

Because the model is empirical, coefficients should be interpreted as fit parameters describing the observed relationship in this dataset, not as physical constants transferable across conditions. However, such high R^2^ values in biological data should be interpreted with caution due to the risk of overfitting. Therefore, we report R^2^ strictly as goodness-of-fit for the descriptive regression, not as evidence of predictive performance (see Section 4.6 and Table S4). For the present dataset, Equation (3) achieved R^2^ = 0.995 (sapphire) and R^2^ = 0.9823 (metal), and we report these values strictly as goodness-of-fit for the descriptive model, not as evidence of predictive performance. The errors between the values predicted by the polynomial function and the experimental values are very close to 0 (see Tables S4 and S5 in the Supplementary Materials).

(3) $$R_{a}\left(\frac{Ca}{P}\right)=a{\left(\frac{Ca}{P}\right)}^{2}+b\left(\frac{Ca}{P}\right)+c\Psi^{2}+d\Psi +e$$

In Equation (3), the coefficients have the following values (for sapphire brackets): a = 0.559243488, b = −1.923881998, c = −1.844730715, d = 7.748247957, e = −2.860790627. A similar procedure was applied to the results obtained for teeth on which metal brackets were bonded. A function with a similar trend to that determined for sapphire brackets was obtained (the function has the same general form, and the terms have the same signs and the same order of magnitude), confirming that the physicochemical process is consistent regardless of the type of bracket. The differences between the coefficients reflect the real variations in the behavior of enamel under the influence of the bracket material. The correlation coefficient R^2^ in this case is 0.9823, indicating that the model explains approximately 98.23% of the variation in surface roughness. The overall model for metal brackets shows a slightly lower correlation coefficient than that for sapphire brackets (0.982 vs. 0.995), but remains highly consistent. In Formula (3), the coefficients have the following values (for metal brackets): a = 0.554969185, b = −1.989780345, c = −0.895096095, d = 3.777313707, e = 0.309852196.

## 4. Discussion

### 4.1. Surface Morphology Evolution

The evolution of the enamel surface morphology over the three experimental stages—before bracket bonding, after bracket removal, and after remineralization—revealed marked structural changes (Figure 8 and Figure 9), consistent with general trends reported for enamel surface behavior in orthodontic procedures [46]. In the present study, the initial enamel surface exhibited a relatively smooth and homogeneous microtopography, reflecting the characteristic structure of sound enamel (Figure 8a and Figure 9a).

Following bracket removal, SEM analysis revealed pronounced surface alterations, including increased surface irregularities, etched enamel regions, and fine microcracks (Figure 5), accompanied by a substantial increase in surface roughness parameters. These morphological changes can be attributed primarily to the combined effects of acid etching and mechanical stresses generated during bracket debonding, all of which are known to compromise the superficial integrity of enamel. After the remineralization step, a partial recovery of the enamel surface morphology was observed. Although the microtopography did not return to the initial pre-bonding state, SEM images indicated a reduction in surface irregularities and a tendency toward a more homogeneous appearance, particularly in the metallic bracket group (Figure 4 and Figure 5). This observation is in agreement with previous reports describing incomplete morphological restoration of enamel following remineralization treatments [47]. Importantly, darker-contrast regions observed in SEM images after debonding and remineralization were interpreted as localized surface depressions and etched enamel areas resulting from topographical variations, rather than as chemically distinct residual materials. The persistence of microstructural heterogeneities after remineralization suggests that this process predominantly involves superficial mineral deposition, leading to chemical recovery (as reflected by Ca/P changes), without complete reconstitution of the original enamel crystal architecture.

### 4.2. Elemental Composition and Ca/P Ratio Dynamics

The elemental analysis in our study revealed significant compositional changes at all stages, underscoring the essential role of calcium and phosphorus in the structural stability of enamel. The Ca/P molar ratio—which theoretically shifts closer to 1.67 for stoichiometric hydroxyapatite—served as a key indicator of mineral integrity [40]. Although stoichiometric hydroxyapatite has a theoretical Ca/P ratio of 1.67, reported Ca/P values for sound human enamel measured by EDX/EDS show a broader distribution (e.g., mean Ca/P around 2.13 ± 0.17, depending on methodology and data reduction). Accordingly, in the present study, EDS-derived Ca/P is used mainly for within-sample, between-stage comparisons rather than as an absolute compositional value. It is important to note that although pure stoichiometric hydroxyapatite has a theoretical ratio of ~1.67, natural human dental enamel is a biological, substituted (e.g., with carbonate ions) apatite that often exhibits deviations from this ideal. Our initial values, although higher, are within the range reported in other in vitro studies for healthy, but non-stoichiometric, enamel [46]. Initial measurements showed high Ca/P ratios, ranging from 1.63 to 2.34 for sapphire brackets and from 1.71 to 2.36 for metal brackets, which may reflect natural enamel heterogeneity and/or methodological dispersion typical for EDS/EDX; therefore, the emphasis is placed on the stage-to-stage change within the same teeth. The primary challenge in interpreting baseline values is the deviation from the theoretical stoichiometric ratio (Ca/P ~ 1.67), which we attribute primarily to the inherent biological heterogeneity of the extracted-teeth sample (different tooth types and donors) and, possibly, to minor methodological artifacts during disinfection.

This initial imbalance could have two main causes, which are not mutually exclusive: 1. Methodological artifact: Although the hydrogen peroxide (10% H_2_O_2_) disinfection protocol was selected for its minimal impact on morphology, we cannot completely exclude the possibility that it induced superficial chemical alterations, such as a preferential loss of phosphate ions compared to calcium ions, thus leading to an artificial increase in the ratio [48,49]. 2. Inherent biological variability: A more plausible explanation is that these values reflect biological heterogeneity of the extracted-teeth sample (different tooth types and donors) rather than an experimental artifact. Because patient-level oral history (diet, fluoride exposure, prior demineralization episodes) was not available, we cannot attribute the baseline Ca/P values to a specific pre-existing condition. Accordingly, we interpret the baseline Ca/P primarily as a reference for within-tooth, between-stage comparisons. Regardless of the cause, this non-stoichiometric starting point is critical for interpreting remineralization data. The critical importance of the Ca/P ratio in hydroxyapatite formation and mineral phase stability has been widely reported in the biomaterials literature, as shown by Wu et al. [50]. This principle also applies to dental tissues, where deviations from ideal stoichiometry may reflect selective ion loss and demineralization processes. Analyses performed in our study after bracket removal revealed a moderate decrease in the Ca/P ratio, with values converging towards the range of 1.85–1.95, suggesting a partial recovery of phosphorus content or removal of residual calcium-rich phases. The most notable compositional changes occurred after remineralization, when the Ca/P ratio decreased further, approaching the theoretical stoichiometry of hydroxyapatite. Final values ranged from 1.70 to 1.91 for metal brackets and from 1.63 to 1.80 for sapphire brackets, indicating a rebalancing of the ionic composition and the formation of a more physiologically relevant mineral phase.

These results suggest that remineralization processes are not only additive in terms of calcium deposition, but involve a more balanced ion incorporation. This interpretation is consistent with the observations reported by Cîrdei et al. [51], who highlighted similar trends in enamel treated with remineralizing agents, with the Ca/P ratio approaching the hydroxyapatite stoichiometry as a sign of structural recovery. This decrease in the Ca/P ratio, which corroborates the increase in absolute concentrations (mentioned in Section 3.1 ), suggests an efficient remineralization mechanism, in which the incorporation of phosphorus was more accelerated than that of calcium, thus compositionally rebalancing the non-stoichiometric enamel.

### 4.3. Correlation Between the Mineral Composition and Surface Roughness

In our study, the statistical correlation between the Ca/P ratio and surface roughness (Ra) demonstrated a clear dependence of enamel topography on mineral composition. Direct correlation attempts using a bivariate function Ra (Ca/P) yielded weak coefficients of determination (R^2^ ~ 0.3), indicating that mineral composition alone cannot fully explain the observed variations in surface roughness. Similar observations have been reported in the literature: SEM–EDS studies have shown that changes in the Ca/P molar ratio during demineralization–remineralization protocols are accompanied by measurable improvements in enamel surface parameters, including a reduction in roughness (Vitiello et al. [47]; Bolty et al. [52]).

In our study, to improve the accuracy of the model, a discrete stage parameter (Ψ) was introduced to capture the distinct physicochemical phenomena associated with each experimental stage (before bonding: Ψ = 1; after bracket removal: Ψ = 2; and post-remineralization: Ψ = 3). The inclusion of this parameter significantly improved the correlation, allowing the development of a second-order polynomial regression model with high descriptive performance/goodness-of-fit (Formula (3)). The function coefficients were of approximately the same order of magnitude, and the functions reached a correlation coefficient of 0.9954 for teeth with sapphire brackets (indicating that the model explained over 99.5% of the roughness variation) and 0.9823 for teeth with metal brackets (indicating that the model explained over 98.23% of the roughness variation). These results highlight a strong nonlinear dependence of the roughness on both mineral composition and treatment stage. The similarity of the functional form and signs of the coefficients between the two types of brackets suggests the existence of a common underlying mechanism, reinforcing the concept that enamel roughness is a direct consequence of compositional changes modulated by physicochemical processes that occur during orthodontic treatment.

### 4.4. Comparative Behavior of Sapphire Versus Metallic Brackets

In our study, a comparative analysis of enamel surface changes associated with sapphire and metallic brackets revealed both common trends and subtle but significant differences in surface and compositional dynamics. Both materials followed a similar evolutionary pattern: an initial state with relatively stable morphology and composition, followed by pronounced demineralization and an increase in roughness after bracket removal, and finally a partial restoration of mineral balance and topographic features after remineralization. This common trajectory supports the hypothesis that the fundamental mechanisms governing the enamel response are independent of the bracket material. Although Sarna-Boś et al. [46] did not investigate the bracket type, their findings similarly highlighted that enamel undergoes comparable compositional and morphological changes during demineralization and remineralization treatments. However, in our study, quantitative differences between the two groups were notable. The Ca/P ratio after remineralization approached slightly closer to the hydroxyapatite stoichiometry in the metal bracket group (1.70–1.91) compared to the sapphire bracket group (1.63–1.80). Furthermore, the regression model for metal brackets yielded a slightly lower correlation coefficient (R^2^ = 0.9823) than that for sapphire brackets (R^2^ = 0.9954), indicating that, although highly predictive, surface roughness in the metal bracket group was influenced by additional factors that were not fully captured by the polynomial function. These differences may be attributed to variations in adhesive bond strength, bracket surface energy, or ion-exchange interactions between the bracket material and the enamel surface. The similarity in sign and magnitude of the regression coefficients for both models further reinforces the conclusion that enamel behavior during orthodontic treatment is governed by a universal set of physicochemical principles. However, the subtle deviations observed suggest that both the composition and surface properties of brackets modulate the kinetics and extent of enamel demineralization and remineralization processes, an aspect that should be taken into account when selecting bracket materials in clinical practice.

### 4.5. Clinical Significance of Surface and Compositional Changes

From a clinical point of view, the observed changes in enamel roughness and composition have significant implications for post-orthodontic treatments and the maintenance of long-term oral health. The increase in surface roughness after bracket removal, associated with microcracks and etching defects, creates a favorable environment for bacterial plaque adhesion and biofilm formation, ultimately increasing the risk of chalky white spots and secondary carious lesions, as shown by Jeon et al. [53]. Furthermore, a rough surface may create discomfort upon contact with the tongue or buccal mucosa and is more susceptible to extrinsic staining, further affecting esthetic perception. Reducing roughness after remineralization—although not restoring enamel to its original state—mitigates these risks by decreasing the surface area available for bacterial colonization. However, it should be emphasized that although this reduction in roughness (from Ra ~3.55 µm to ~2.11 µm in the sapphire group) is statistically significant, the final values remain almost ten times higher than the clinically accepted threshold (Ra < 0.2 µm) for preventing plaque retention [34]. It is important to note that the roughness values reported here (Ra ~ 1.7–3.5 µm) were derived from SEM images processed by 3D Roughness Reconstruction using the shape-from-shading algorithm. This methodology captures the microtopography of the entire scanned area, often yielding higher absolute Ra values compared to traditional 2D stylus profilometry, which was predominantly used in the literature (e.g., Bollen et al.) to establish the clinically accepted Ra < 0.2 µm threshold for bacterial retention [34]. Despite this methodological difference in absolute values, the relative finding remains critically relevant: the enamel surface after the short-term remineralization protocol remains highly irregular (Ra ~ 2.11 µm in the sapphire group) and significantly rougher than the initial baseline (Ra ~ 1.4 µm). Therefore, the clinical verdict that the surface remains in an unfavorable state for plaque retention is maintained, regardless of the absolute value difference arising from the measurement technique. Therefore, although a useful step, remineralization therapy in the form applied here failed to restore the surface to a favorable clinical state. Furthermore, the progressive normalization of the Ca/P ratio towards the theoretical value of hydroxyapatite (~1.67) suggests a recovery of the physicochemical properties of the enamel and its resistance to acid attack. This compositional rebalancing is essential for increasing enamel durability and reducing susceptibility to demineralization in the post-orthodontic period. The regression models developed in this study provide a descriptive framework for linking enamel composition (Ca/P) and surface roughness (Ra) within the present in vitro dataset. Their use as predictive tools would require external validation on independent samples and, ideally, in vivo confirmation.

### 4.6. Validation and Robustness of the Mathematical Model

Within the present in vitro dataset, the polynomial regression model provided a very good descriptive fit for both bracket types (R^2^ = 0.9954 for sapphire and R^2^ = 0.9823 for metal). The model indicates that enamel condition in our experiment can be summarized through a combined relationship between the surface roughness (Ra), the ratio, and the stage parameter (Ψ). Crucially, the exceptionally high descriptive power (R^2^ > 0.98) was only achieved after incorporating the discrete stage parameter (Ψ), demonstrating that the relationship between the surface roughness and the Ca/P ratio is fundamentally dependent on the physicochemical processes specific to each experimental stage.

In biological datasets, very high R^2^ values may reflect severe overfitting, meaning that the model captures sample-specific and potentially artifactual variability that severely limits the model’s generalizability. Therefore, the model’s strength is purely descriptive within this confined in vitro dataset.

Although residual inspection did not indicate an obvious systematic bias for the fitted data, the actual predictive accuracy on new teeth (external validation) and under in vivo conditions remains unknown and must be tested in future work.

Accordingly, we present the model primarily as a descriptive tool that integrates compositional (EDS) and morphological (roughness) outputs in a unified framework, while acknowledging that its clinical or predictive use requires independent validation.

These R^2^ values indicate that, within this dataset, more than 98% of the variability in Ra is explained by Ca/P and the stage parameter (Ψ). This type of modeling has been discussed in the broader context of dental materials research (Paolone et al. [54]).

### 4.7. Study Limitations

Despite the good descriptive fit of the regression model and the comprehensive experimental design, several limitations should be acknowledged. First, the study was conducted under controlled laboratory conditions, which may not fully reproduce the complex oral environment, including variations in salivary composition, pH fluctuations, and masticatory forces. Future studies should aim to validate the proposed models in vivo to assess their predictive accuracy under physiological conditions. The current model (describing the Ra (Ca/P) function) is based on a second-order polynomial that, although highly efficient, may not fully capture complex nonlinear interactions. Future research could explore machine learning-based methods or multivariate approaches to develop more sophisticated predictive models capable of integrating additional variables. The study also deliberately used a heterogeneous collection of tooth types (incisors, canines, premolars, and molars) to simulate a variety of clinically encountered enamel surfaces. We recognize that these teeth differ in anatomy and enamel thickness, which is a confounding factor. Although random assignment and a very large sample size (N = 200) were used to statistically distribute this variability, this approach does not eliminate it. The justification for this choice is twofold: (1) it reflects a pragmatic approach related to the difficulty of obtaining such a large sample size from a single tooth type (e.g., 200 identical premolars) and (2) the primary objective was to establish a general correlation pattern (Ra vs. Ca/P), not to quantify damage on a specific tooth type. However, we admit that a subgroup analysis (e.g., comparing only premolars from the sapphire group with those from the metal group) is lacking and could provide valuable additional data, and this remains a direction for future research. We specifically acknowledge that the reported differences between sapphire and metallic brackets (e.g., in mineral loss and roughness magnitude) represent the average statistical trend across the mixed tooth sample. Consequently, our findings should be interpreted cautiously, as they may not accurately reflect the difference in damage severity on a single, specific tooth type (e.g., a molar vs. an incisor).

As discussed in Section 4.2, a central limitation is the initial composition of the sample. The Ca/P ratio values (often >2.0) deviated significantly from the ideal stoichiometric value of 1.67 (for hydroxyapatite). This suggests that the base sample was not compositionally ideal, either due to biological variability (e.g., teeth with a periodontal history) or as a possible artifact of disinfection. This non-stoichiometric starting point complicates the interpretation of the remineralization results. Although we observed a tendency to ‘reequilibrate’ towards 1.67 after treatment, it is not clear whether this represents optimal structural restoration or simply mineral precipitation on an already compromised substrate. The observed rates of demineralization and remineralization could have been directly influenced by this initial state of non-stoichiometric enamel/inter-sample variability. Third, the remineralization protocol was limited to a duration of 10 days, simulating a short-term clinical intervention. Our results, which indicate only partial recovery, emphasize that this study cannot assess the long-term efficacy or stability of the remineralized layer over time. Future studies are needed to determine whether prolonged treatment could lead to complete enamel restoration. Second, the study evaluated a single remineralization agent (GC Tooth Mousse^®^ with fluoride). The choice of this product was based on its widespread clinical use, and the application protocol (3 min/day for 10 days) was not arbitrary, but strictly followed the manufacturer’s instructions for a short-term clinical regimen, as detailed in Section 2.8 [55].

However, we acknowledge that this is a limitation. The findings on remineralization efficacy are specific to this product and protocol. The study did not discuss or compare the efficacy of other modern agents (e.g., those based on simple CPP-ACP, bioactive glass, or nano-hydroxyapatite), which may provide different results. A comparative analysis of these agents remains an important direction for future research.

In addition, the experimental design was focused on isolating the effect of the bracket material (sapphire vs. metal). Therefore, all other factors, such as the adhesive agent (Transbond™ XT) and the etching method, were deliberately kept constant. This controlled approach necessarily means that our study cannot provide information about the complex interactions or cumulative effects that might occur when using other adhesive systems or etching techniques, which remain a topic for future investigation.

Despite these limitations, the results of this study provide valuable information about the interaction between mineral composition, surface roughness, and treatment stage. They provide a solid foundation for future research aimed at optimizing orthodontic materials, refining remineralization strategies, and improving clinical outcomes for patients.

## 5. Conclusions

The enamel surfaces showed a substantial increase in roughness and microstructural degradation after bracket removal, manifested by microcracks, irregular etching patterns, and residual adhesive traces. At the beginning of the experiment, no statistically significant differences were found between the sapphire and metallic bracket groups. Following bracket bonding and removal, however, significant differences emerged, with sapphire brackets leading to higher mean roughness and mineral loss values. Based on the observed statistical average of the mixed sample, these quantitative differences suggest that the bracket type influences the extent of enamel alteration and its subsequent recovery. After remineralization, the differences diminished but remained detectable, indicating that the type of bracket influences the extent of enamel alteration and its subsequent recovery. The application of remineralizing agents favored a partial statistical recovery and smoothing of the surface; however, the initial microarchitecture was not restored. Crucially, the final roughness values (Ra ≈ 1.7–2.1 µm) remain significantly above the clinical threshold of 0.2 µm, indicating that the enamel surface is still in an unfavorable state and susceptible to plaque retention.

The results of the study emphasize that although post-orthodontic remineralization therapies are important, the short-term protocol tested was insufficient to achieve a safe clinical state. This fact emphasizes the need for personalized, much longer or more advanced treatment strategies adapted to the enamel type to properly manage the damage. It should be strongly emphasized that the quantitative conclusions regarding the comparative performance (sapphire vs. metallic) are based on the average statistical response of the mixed sample. Future research is critically needed to validate whether these differences hold and are clinically relevant within homogeneous dental subgroups.

A mathematical model based on polynomial regression was developed, which successfully describes the correlation of roughness (Ra) with the Ca/P ratio only when incorporating the experimental stage parameter (Ψ). This model emphasizes that the strong statistical relationship between surface roughness and mineral composition is fundamentally stage-dependent.

Although the model demonstrated exceptional descriptive capacity of in vitro data (R^2^ > 0.98), the biological relevance of this high R^2^ value is severely limited by the risk of overfitting. Therefore, its predictive value is currently restricted, and rigorous external validation is mandatory before this model can be tested as a potential diagnostic tool.

## Figures and Tables

**Table 1:** Mean atomic percentages (at.%) of calcium (Ca) and phosphorus (P) (mean ± SD) determined by EDS for the two bracket types across all experimental stages.

Bracket Type	Stage	Ca (%)	P (%)
Sapphire	Stage 1 (Initial)	32.65 ± 1.7	16.35 ± 0.9
Sapphire	Stage 2 (Post-debonding)	16.37 ± 0.85	8.60 ± 0.3
Sapphire	Stage 3 (Post-remineralization)	23.58 ± 0.85	12.8 ± 0.56
Metallic	Stage 1 (Initial)	32.96 ± 1.86	16.48 ± 0.88
Metallic	Stage 2 (Post-debonding)	18.52 ± 0.84	9.42 ± 0.32
Metallic	Stage 3 (Post-remineralization)	25.86 ± 0.56	14.56 ± 0.28

Note: n = 100 teeth per group; for each tooth, values represent the average of 14 measurement points within the ROI. Ca—calcium; P—phosphorus; SD—standard deviation; at.%—atomic percentage.

## Data Availability

The minimum necessary raw data and derived statistical tables supporting the reported findings are available within the Supplementary Materials. Further detailed datasets (e.g., the complete set of individual measurement points) are available from the corresponding authors upon reasonable request.

## Supplementary Materials

The following supporting information can be downloaded at https://www.mdpi.com/article/10.3390/bioengineering13010085/s1. Table S1. Power statistics results for Ca and P atomic concentration “Mean ± SD (at.%)”. Table S2. Kolmogorov-Smirnov test for Ca and P atomic concentration. Table S3. Games-Howell test Ca and P atomic concentration. Table S4. The polynomial function data obtained by regression analysis for teeth with sapphire brackets. Table S5. The polynomial function data obtained by regression analysis for teeth with metallic brackets.
