# Supplementary material for: Integrated Characterization by EDS and Roughness as a Diagnostic Tool for Dental Enamel Degradation: An In Vitro Study
Source: Bioengineering (Basel). 2026 Jan 12;13(1):85. doi: 10.3390/bioengineering13010085 (PMC12837259; doi:10.3390/bioengineering13010085)
Supplement: Supplementary file 1 [file bioengineering-13-00085-s001.zip › bioengineering-4024428-supplementary.pdf]

Table S1. Power statistics results for Ca and P atomic concentration „Mean ± SD (at.%)".

Element	Sapphire	Specimens (teeth)	Mean difference	Standard deviation of difference	Cohen's factor	Power
Ca	Initial vs debracketing	100	16.5267	1.921281	8.601919	1
	Debracketing vs remineralization	100	-7.2548	1.151804	-6.29864	1
	Initial vs remineralization	100	9.2719	2.098399	4.418559	1
P	Initial vs debracketing	100	8.0192	0.963551	8.322552	1
	Debracketing vs remineralization	100	-4.5285	0.62527	-7.24247	1
	Initial vs remineralization	100	3.4907	1.032053	3.382287	1
**Element**	**Metallic**	**Specimens (teeth)**	**Mean difference**	**Standard deviation of difference**	**Cohen's factor**	**Power**
Ca	Initial vs debracketing	100	14.41	2.15	6.72	1
	Debracketing vs remineralization	100	6.94	1.89	3.67	1
	Initial vs remineralization	100	-7.47	1.07	-6.98	1
P	Initial vs debracketing	100	7.00	0.93	7.52	1
	Debracketing vs remineralization	100	2.01	0.98	2.05	1
	Initial vs remineralization	100	-4.99	0.37	-13.32	1

Table S2. Kolmogorov-Smirnov test for Ca and P atomic concentration.

Element		Specimens (teeth)	D	p	α	Bracket
Ca	Initial	100	0.0758	0.5863	0.01	Sapphire
	Debracketing	100	0.0987	0.2658		
	Remineralization	100	0.1109	0.1588		
P	Initial	100	0.1118	0.1521		
	Debracketing	100	0.0943	0.3166		
	Remineralization	100	0.1108	0.1577		
Ca	Initial	100	0.1030	0.2229		Metallic
	Debracketing	100	0.0911	0.3565		
	Remineralization	100	0.0964	0.2920		
P	Initial	100	0.0881	0.3962		
	Debracketing	100	0.0957	0.2997		
	Remineralization	100	0.1068	0.1900		

Table S3. Games-Howell test Ca and P atomic concentration.

Element	Sapphire	Specimens (teeth)	Mean difference	95% Confidence Interval	p-value
Ca	Initial vs debracketing	100	17.20	[16.65 – 17.75]	<0.001

	Debracketing vs remineralization	100	10.08	[9.45 – 10.72]	<0.001
	Initial vs remineralization	100	7.12	[6.54 – 7.70]	<0.001
P	Initial vs debracketing	100	8.22	[8.00 – 8.44]	<0.001
	Debracketing vs remineralization	100	3.68	[3.45 – 3.91]	<0.001
	Initial vs remineralization	100	4.54	[4.32 – 4.76]	<0.001
**Element**	**Metallic**	**Specimens (teeth)**	**Mean difference**	**95% Confidence Interval**	**p-value**
Ca	Initial vs debracketing	100	14.41	[13.94 – 14.88]	<0.001
	Debracketing vs remineralization	100	6.94	[6.51 – 7.38]	<0.001
	Initial vs remineralization	100	−7.47	[−7.72 – −7.22]	<0.001
P	Initial vs debracketing	100	7.0022	[6.7831–7.2213]	<0.001
	Debracketing vs remineralization	100	2.0070	[1.7866–2.2274]	<0.001
	Initial vs remineralization	100	−4.9952	[−5.0913–−4.8991]	<0.001

Table S4. The polynomial function data obtained by regression analysis for teeth with sapphire brackets.

Ratio Ca/P	Ra [μm]	Phase Ψ	The value predicted by the polynomial function	The difference between the experimental value and the calculated value
1.90	1.41	1	1.40722	0.00478
2.34	1.59	1	1.603719	-0.01872
2.22	1.52	1	1.528767	-0.00877
1.90	1.48	1	1.405768	0.074232
1.84	1.40	1	1.396088	0.003912
2.06	1.35	1	1.452185	-0.10519
1.98	1.32	1	1.42697	-0.10997
2.07	1.56	1	1.457302	0.102698
2.24	1.48	1	1.537947	-0.05795
2.21	1.51	1	1.52374	-0.01174
1.81	1.31	1	1.392539	-0.08654
2.10	1.59	1	1.467648	0.123352
2.06	1.55	1	1.453915	0.096085
2.00	1.36	1	1.430549	-0.06655
1.81	1.36	1	1.393037	-0.03804
1.98	1.36	1	1.425791	-0.07079
1.99	1.39	1	1.429427	-0.03843
1.92	1.46	1	1.411111	0.045889
1.92	1.43	1	1.411079	0.018921
1.81	1.39	1	1.392549	-0.00555

1.98	1.48	1	1.427097	0.056903
1.85	1.34	1	1.397083	-0.05508
2.08	1.39	1	1.459712	-0.07171
2.00	1.41	1	1.431937	-0.02194
2.07	1.44	1	1.457438	-0.02044
2.21	1.54	1	1.520498	0.015502
1.74	1.36	1	1.388362	-0.02836
2.05	1.45	1	1.447201	0.006799
1.90	1.48	1	1.405745	0.072255
1.79	1.31	1	1.391071	-0.07707
1.94	1.48	1	1.414222	0.067778
1.88	1.35	1	1.402216	-0.05122
1.82	1.32	1	1.393315	-0.07331
2.17	1.59	1	1.499149	0.085851
2.30	1.59	1	1.573419	0.016581
2.03	1.54	1	1.44173	0.10127
2.01	1.39	1	1.434952	-0.04395
2.03	1.33	1	1.441784	-0.11278
2.01	1.51	1	1.436291	0.068709
2.10	1.43	1	1.469579	-0.03758
1.72	1.34	1	1.388126	-0.05113
1.85	1.45	1	1.396987	0.052013
1.70	1.31	1	1.38828	-0.07828
2.20	1.57	1	1.517525	0.055475
2.10	1.38	1	1.467293	-0.08929
1.91	1.50	1	1.40853	0.09047
1.96	1.39	1	1.419671	-0.02567
1.85	1.46	1	1.397596	0.058404
1.86	1.46	1	1.399105	0.064895
1.77	1.36	1	1.389602	-0.0346
2.26	1.59	1	1.548539	0.042461
2.14	1.53	1	1.488705	0.044295
2.03	1.58	1	1.4421	0.1399
2.22	1.57	1	1.52652	0.04148
2.17	1.48	1	1.499171	-0.02017
2.13	1.58	1	1.482721	0.094279
1.71	1.33	1	1.388135	-0.06113
1.82	1.36	1	1.394209	-0.03521
1.81	1.31	1	1.392789	-0.07879
2.09	1.40	1	1.46448	-0.06648
1.92	1.42	1	1.410177	0.006823
1.76	1.38	1	1.389014	-0.00801
2.27	1.55	1	1.555911	-0.00691

1.94	1.41	1	1.415657	-0.00866
1.80	1.38	1	1.391468	-0.00747
1.93	1.46	1	1.413108	0.049892
1.81	1.34	1	1.392163	-0.05016
2.03	1.54	1	1.443398	0.097602
1.89	1.32	1	1.404966	-0.08297
2.26	1.60	1	1.552334	0.043666
1.99	1.53	1	1.427903	0.104097
1.79	1.36	1	1.390807	-0.03081
1.71	1.30	1	1.388222	-0.08622
1.97	1.55	1	1.422142	0.122858
2.07	1.51	1	1.457137	0.054863
2.08	1.52	1	1.461788	0.057212
1.99	1.53	1	1.429278	0.101722
1.80	1.32	1	1.391328	-0.06933
1.88	1.41	1	1.402244	0.005756
1.77	1.34	1	1.389263	-0.05426
1.99	1.56	1	1.429147	0.129853
2.11	1.49	1	1.472028	0.014972
1.98	1.40	1	1.426852	-0.02785
1.99	1.32	1	1.428326	-0.10933
1.90	1.39	1	1.407232	-0.01423
2.11	1.40	1	1.475115	-0.07711
2.10	1.52	1	1.467291	0.051709
2.03	1.49	1	1.443325	0.047675
2.23	1.57	1	1.531818	0.034182
1.96	1.44	1	1.419683	0.022317
2.04	1.34	1	1.443857	-0.10786
2.27	1.51	1	1.556439	-0.04244
1.98	1.53	1	1.425391	0.102609
2.08	1.47	1	1.458663	0.009337
2.25	1.53	1	1.546127	-0.01513
1.99	1.45	1	1.428601	0.019399
1.91	1.46	1	1.409255	0.047745
2.08	1.43	1	1.461027	-0.03303
1.79	1.31	1	1.390635	-0.08264
2.01	1.33	1	1.434644	-0.10264
1.89	3.66	2	3.619044	0.041956
1.85	3.52	2	3.612322	-0.09132
1.75	3.54	2	3.602701	-0.0627
1.75	3.73	2	3.602644	0.122356
1.74	3.55	2	3.602477	-0.05248
1.89	3.50	2	3.618597	-0.1166

1.80	3.53	2	3.605521	-0.08052
1.82	3.67	2	3.607543	0.058457
2.00	3.50	2	3.646373	-0.14537
2.01	3.54	2	3.649374	-0.10937
1.84	3.64	2	3.610136	0.026864
1.94	3.67	2	3.628121	0.044879
1.89	3.66	2	3.617968	0.045032
1.90	3.56	2	3.619631	-0.06363
2.02	3.68	2	3.653477	0.024523
1.99	3.56	2	3.642556	-0.08356
1.86	3.58	2	3.612465	-0.03146
1.85	3.69	2	3.611201	0.075799
1.95	3.66	2	3.63249	0.02951
2.15	3.71	2	3.704077	0.007923
1.79	3.66	2	3.604703	0.059297
1.88	3.64	2	3.61704	0.02496
1.81	3.52	2	3.606305	-0.08331
1.83	3.59	2	3.609416	-0.01742
1.88	3.57	2	3.617344	-0.05134
1.90	3.56	2	3.620682	-0.05968
1.85	3.74	2	3.611595	0.131405
1.95	3.60	2	3.631794	-0.03379
1.82	3.72	2	3.607329	0.115671
1.78	3.66	2	3.604516	0.053484
2.13	3.70	2	3.696991	0.002009
2.06	3.63	2	3.665647	-0.03965
2.01	3.64	2	3.649089	-0.00509
2.04	3.62	2	3.660973	-0.03797
1.90	3.55	2	3.619487	-0.07049
1.88	3.68	2	3.616147	0.064853
1.99	3.57	2	3.643041	-0.07304
1.80	3.51	2	3.606101	-0.1001
1.96	3.66	2	3.634125	0.026875
1.94	3.54	2	3.628902	-0.0849
1.76	3.74	2	3.603224	0.131776
1.82	3.74	2	3.607407	0.130593
1.88	3.73	2	3.616226	0.112774
1.85	3.59	2	3.611857	-0.01886
1.73	3.50	2	3.602205	-0.0982
2.05	3.73	2	3.663002	0.068998
2.04	3.61	2	3.659012	-0.05201
2.12	3.74	2	3.693726	0.048274
1.96	3.74	2	3.633905	0.107095

2.06	3.71	2	3.66579	0.04721
1.72	3.57	2	3.602171	-0.02817
1.95	3.60	2	3.631032	-0.03503
1.87	3.71	2	3.615546	0.097454
1.96	3.58	2	3.63412	-0.05512
1.81	3.54	2	3.606694	-0.06469
1.84	3.64	2	3.610473	0.028527
1.81	3.73	2	3.606426	0.127574
1.92	3.67	2	3.623524	0.050476
2.00	3.64	2	3.645257	-0.00226
1.94	3.52	2	3.62831	-0.10431
1.81	3.65	2	3.606869	0.047131
2.00	3.75	2	3.645992	0.102008
1.92	3.54	2	3.624057	-0.08906
1.87	3.63	2	3.614047	0.015953
1.82	3.72	2	3.607566	0.111434
1.77	3.69	2	3.603503	0.081497
1.91	3.67	2	3.622385	0.051615
1.91	3.68	2	3.62208	0.05392
1.86	3.59	2	3.613671	-0.02367
1.91	3.57	2	3.623018	-0.05002
1.87	3.70	2	3.614721	0.087279
2.15	3.70	2	3.705276	-0.00228
1.90	3.72	2	3.619386	0.097614
2.07	3.73	2	3.669125	0.058875
1.81	3.63	2	3.606237	0.021763
2.13	3.63	2	3.695825	-0.07082
1.83	3.70	2	3.608997	0.091003
1.87	3.66	2	3.613951	0.048049
2.18	3.68	2	3.719868	-0.04487
1.97	3.70	2	3.636126	0.062874
1.99	3.72	2	3.641484	0.081516
1.95	3.58	2	3.631561	-0.04756
1.84	3.59	2	3.610491	-0.01649
1.92	3.52	2	3.623538	-0.10054
1.99	3.65	2	3.642897	0.002103
1.97	3.51	2	3.63658	-0.12758
1.94	3.62	2	3.629409	-0.01341
1.94	3.64	2	3.628926	0.007074
1.93	3.57	2	3.625765	-0.05376
1.89	3.65	2	3.618442	0.029558
1.79	3.51	2	3.604628	-0.09663
1.88	3.51	2	3.616849	-0.10785

1.92	3.71	2	3.624234	0.081766
1.85	3.59	2	3.611382	-0.02138
1.86	3.53	2	3.613742	-0.08174
2.06	3.63	2	3.665828	-0.03483
1.93	3.69	2	3.626971	0.065029
2.04	3.55	2	3.660835	-0.10684
1.76	3.66	2	3.6031	0.0529
1.97	3.52	2	3.636786	-0.11579
1.62	2.08	3	2.132645	-0.04965
1.67	2.19	3	2.128217	0.058783
1.74	2.14	3	2.126978	0.009022
1.92	2.18	3	2.149784	0.027216
1.89	2.11	3	2.14272	-0.03072
1.73	2.19	3	2.126805	0.059195
1.84	2.12	3	2.135024	-0.01402
1.97	2.07	3	2.162862	-0.09186
1.92	2.19	3	2.149996	0.038004
2.02	2.08	3	2.17587	-0.09387
1.70	2.11	3	2.127033	-0.01503
1.65	2.19	3	2.129423	0.064577
1.82	2.19	3	2.132398	0.061602
1.84	2.15	3	2.134992	0.010008
1.94	2.15	3	2.153806	-0.00181
1.78	2.13	3	2.128759	-0.00076
1.76	2.11	3	2.127526	-0.01953
1.65	2.11	3	2.129347	-0.01635
1.76	2.16	3	2.127797	0.029203
1.94	2.17	3	2.154701	0.013299
1.85	2.17	3	2.136107	0.036893
1.92	2.17	3	2.149471	0.023529
1.67	2.08	3	2.128061	-0.04606
1.66	2.13	3	2.129003	0.004997
1.88	2.08	3	2.140732	-0.06373
1.91	2.14	3	2.146219	-0.00522
1.82	2.13	3	2.132578	-0.00558
1.78	2.19	3	2.129005	0.055995
1.76	2.12	3	2.127605	-0.01161
1.95	2.09	3	2.15735	-0.07235
1.67	2.09	3	2.127987	-0.03899
1.78	2.17	3	2.129057	0.039943
1.78	2.15	3	2.129011	0.020989
1.78	2.08	3	2.128628	-0.04563
1.65	2.08	3	2.129825	-0.04883

1.66	2.16	3	2.128807	0.032193
1.87	2.08	3	2.139251	-0.06025
1.90	2.18	3	2.145157	0.031843
1.63	2.16	3	2.131172	0.030828
1.93	2.08	3	2.151251	-0.07025
1.96	2.08	3	2.157698	-0.0767
1.73	2.20	3	2.126852	0.071148
1.86	2.12	3	2.138496	-0.0195
1.76	2.12	3	2.127816	-0.00982
1.77	2.18	3	2.128219	0.047781
1.81	2.19	3	2.130827	0.062173
1.97	2.20	3	2.160952	0.037048
1.98	2.17	3	2.16403	0.00397
1.80	2.12	3	2.130629	-0.01163
1.81	2.08	3	2.131251	-0.05025
1.87	2.17	3	2.139125	0.031875
1.75	2.14	3	2.12726	0.01574
1.79	2.13	3	2.1298	-0.0048
1.73	2.19	3	2.126844	0.061156
1.60	2.08	3	2.135361	-0.05136
1.98	2.13	3	2.165485	-0.03148
1.78	2.07	3	2.128886	-0.05789
1.86	2.13	3	2.137701	-0.0067
1.66	2.08	3	2.129083	-0.05208
1.78	2.09	3	2.128881	-0.04388
1.71	2.09	3	2.126815	-0.04181
1.80	2.15	3	2.130285	0.023715
1.81	2.17	3	2.131019	0.035981
1.83	2.15	3	2.133668	0.012332
1.79	2.20	3	2.129465	0.065535
1.60	2.12	3	2.13534	-0.01634
2.06	2.11	3	2.192161	-0.08516
1.88	2.18	3	2.141649	0.041351
2.01	2.10	3	2.172915	-0.07392
1.81	2.20	3	2.131329	0.063671
1.74	2.07	3	2.126936	-0.05494
1.95	2.20	3	2.155949	0.040051
1.89	2.08	3	2.143669	-0.06767
1.70	2.19	3	2.127073	0.058927
1.71	2.14	3	2.126876	0.012124
1.83	2.20	3	2.134134	0.064866
1.85	2.08	3	2.136618	-0.05662
1.87	2.14	3	2.138608	0.003392

1.94	2.20	3	2.152771	0.043229
1.78	2.14	3	2.128473	0.009527
1.83	2.15	3	2.133202	0.018798
1.63	2.16	3	2.131696	0.028304
1.78	2.13	3	2.128822	0.000178
1.63	2.15	3	2.131435	0.020565
1.86	2.15	3	2.138422	0.007578
1.79	2.19	3	2.12934	0.05766
1.93	2.08	3	2.151243	-0.07524
1.93	2.11	3	2.151116	-0.04412
1.72	2.19	3	2.126769	0.067231
1.67	2.19	3	2.128154	0.057846
1.86	2.13	3	2.137947	-0.00895
1.81	2.15	3	2.130919	0.020081
1.67	2.11	3	2.128303	-0.0223
1.79	2.09	3	2.129539	-0.03554
1.82	2.13	3	2.13193	-0.00193
1.78	2.12	3	2.128892	-0.01289
1.80	2.15	3	2.130656	0.015344
1.89	2.08	3	2.142788	-0.06279
1.82	2.20	3	2.132419	0.064581
1.81	2.20	3	2.1315	0.0665

Table S5. The polynomial function data obtained by regression analysis for teeth with metallic brackets.

**Ratio Ca/P**	**Ra [μm]**	**Phase Ψ**	**The value predicted by the polynomial function**	**The difference between the experimental value and the calculated value**
1.99	1.31	1	1.430454	-0.12145
2.04	1.49	1	1.442088	0.048912
1.92	1.39	1	1.417357	-0.02336
1.95	1.45	1	1.423059	0.029941
2.06	1.35	1	1.449218	-0.09922
1.76	1.38	1	1.409249	-0.03425
1.96	1.42	1	1.424318	-0.00132
2.16	1.53	1	1.4843	0.0427
1.80	1.37	1	1.408606	-0.03961
1.93	1.32	1	1.418491	-0.09549
1.94	1.39	1	1.421384	-0.03438
2.03	1.35	1	1.440358	-0.09236
2.36	1.58	1	1.586777	-0.00778
1.95	1.54	1	1.421924	0.120076
1.93	1.49	1	1.419048	0.070952
2.06	1.56	1	1.448618	0.112382

2.15	1.54	1	1.477578	0.063422
2.01	1.36	1	1.433587	-0.07759
2.29	1.57	1	1.543431	0.024569
2.11	1.46	1	1.464579	-0.00258
2.09	1.54	1	1.458416	0.083584
2.06	1.57	1	1.449439	0.119561
1.88	1.40	1	1.412695	-0.0177
1.94	1.33	1	1.41987	-0.08687
1.76	1.37	1	1.409265	-0.04127
1.89	1.43	1	1.414	0.014
2.10	1.55	1	1.459401	0.085599
2.09	1.56	1	1.456895	0.101105
1.85	1.30	1	1.410201	-0.1082
2.10	1.45	1	1.461075	-0.00808
2.02	1.43	1	1.437819	-0.01282
1.81	1.37	1	1.408791	-0.04179
2.04	1.34	1	1.443182	-0.10718
2.09	1.40	1	1.456489	-0.05549
2.36	1.58	1	1.584229	-0.00123
2.11	1.40	1	1.465272	-0.06827
1.88	1.46	1	1.413209	0.042791
2.00	1.51	1	1.432386	0.078614
1.96	1.41	1	1.424034	-0.01503
2.34	1.59	1	1.576821	0.015179
2.17	1.59	1	1.488308	0.100692
1.92	1.38	1	1.417389	-0.04139
2.13	1.45	1	1.469819	-0.02082
1.95	1.39	1	1.422538	-0.03254
1.96	1.39	1	1.423592	-0.03859
1.79	1.31	1	1.408536	-0.09754
1.99	1.48	1	1.430258	0.052742
2.18	1.45	1	1.492205	-0.0412
1.88	1.32	1	1.412792	-0.09779
1.88	1.38	1	1.412404	-0.0284
2.15	1.57	1	1.478275	0.093725
1.79	1.37	1	1.408542	-0.03654
1.82	1.34	1	1.408892	-0.06589
2.13	1.45	1	1.470386	-0.02339
2.36	1.60	1	1.587741	0.008259
1.86	1.37	1	1.410877	-0.03788
2.26	1.50	1	1.527986	-0.02599
2.06	1.53	1	1.448979	0.079021
1.76	1.37	1	1.409001	-0.038

2.05	1.52	1	1.446544	0.071456
1.99	1.41	1	1.430845	-0.02085
2.00	1.49	1	1.431444	0.058556
2.06	1.49	1	1.449612	0.040388
2.00	1.46	1	1.431428	0.029572
1.71	1.33	1	1.41199	-0.08499
2.17	1.55	1	1.486065	0.064935
1.79	1.40	1	1.408567	-0.01257
1.76	1.36	1	1.409275	-0.05327
1.94	1.31	1	1.42053	-0.10853
2.21	1.48	1	1.503238	-0.02624
2.23	1.50	1	1.513117	-0.01012
2.00	1.31	1	1.432386	-0.12739
2.16	1.45	1	1.485158	-0.03116
1.84	1.37	1	1.409741	-0.04174
2.23	1.49	1	1.51313	-0.01913
1.95	1.35	1	1.421492	-0.06949
1.95	1.51	1	1.421937	0.085063
2.14	1.42	1	1.477284	-0.06128
2.04	1.58	1	1.443148	0.137852
1.95	1.34	1	1.421428	-0.08043
2.09	1.40	1	1.456915	-0.05491
1.80	1.33	1	1.408539	-0.07454
2.10	1.58	1	1.462594	0.114406
2.00	1.56	1	1.432386	0.130614
1.83	1.38	1	1.409448	-0.03245
1.95	1.50	1	1.422374	0.075626
2.20	1.55	1	1.501462	0.043538
2.15	1.47	1	1.477876	-0.01088
1.92	1.46	1	1.418028	0.040972
1.81	1.37	1	1.408625	-0.03563
1.70	1.33	1	1.413241	-0.08524
2.18	1.57	1	1.491171	0.077829
2.20	1.57	1	1.498818	0.071182
1.95	1.49	1	1.422334	0.067666
2.00	1.40	1	1.432242	-0.03024
1.80	1.41	1	1.408612	-0.00361
1.95	1.52	1	1.423064	0.094936
2.17	1.57	1	1.488332	0.080668
2.05	1.57	1	1.444578	0.121422
2.01	1.53	1	1.434567	0.099433
2.07	2.46	2	2.544087	-0.08609
2.06	2.53	2	2.540485	-0.01048

1.95	2.53	2	2.514851	0.016149
2.03	2.55	2	2.531611	0.014389
2.02	2.49	2	2.529817	-0.03982
1.77	2.60	2	2.500887	0.095113
2.01	2.53	2	2.526567	0.000433
2.08	2.50	2	2.547844	-0.04984
2.14	2.57	2	2.568908	9.18E-05
2.04	2.49	2	2.535827	-0.04483
1.96	2.52	2	2.516951	-0.00095
1.97	2.46	2	2.517756	-0.05576
2.06	2.45	2	2.539686	-0.08569
2.08	2.59	2	2.547297	0.046703
1.79	2.58	2	2.500564	0.074436
1.81	2.55	2	2.500715	0.053285
1.95	2.51	2	2.514568	-0.00357
1.95	2.48	2	2.514781	-0.03878
2.05	2.47	2	2.535912	-0.06291
1.91	2.49	2	2.508093	-0.02009
1.90	2.53	2	2.507281	0.024719
1.91	2.56	2	2.508393	0.048607
2.01	2.55	2	2.527532	0.021468
1.99	2.49	2	2.522796	-0.0308
2.07	2.59	2	2.542908	0.050092
2.04	2.56	2	2.534784	0.026216
1.97	2.53	2	2.517546	0.015454
1.87	2.54	2	2.503702	0.038298
1.78	2.51	2	2.500614	0.012386
2.12	2.49	2	2.558409	-0.07141
2.13	2.50	2	2.562553	-0.05955
2.03	2.56	2	2.533034	0.030966
2.00	2.45	2	2.525108	-0.07311
1.96	2.47	2	2.515924	-0.04892
2.11	2.46	2	2.554961	-0.09796
2.14	2.46	2	2.567178	-0.11118
2.17	2.58	2	2.578484	-0.00048
1.79	2.56	2	2.500565	0.055435
2.01	2.52	2	2.527267	-0.00627
1.82	2.47	2	2.500843	-0.03584
2.14	2.52	2	2.568429	-0.04443
1.97	2.52	2	2.518142	0.002858
1.83	2.48	2	2.50147	-0.02547
1.93	2.52	2	2.511783	0.003217
2.05	2.51	2	2.536364	-0.02636

2.01	2.54	2	2.525705	0.016295
1.90	2.55	2	2.506617	0.038383
1.92	2.46	2	2.509584	-0.05258
1.88	2.51	2	2.504835	0.001165
2.01	2.54	2	2.527374	0.016626
1.93	2.53	2	2.511538	0.013462
2.02	2.58	2	2.529933	0.048067
2.03	2.55	2	2.532523	0.016477
1.88	2.47	2	2.505286	-0.03129
2.08	2.46	2	2.547611	-0.08661
1.94	2.55	2	2.512828	0.033172
1.83	2.45	2	2.501242	-0.04724
1.89	2.54	2	2.505644	0.032356
2.10	2.59	2	2.553455	0.037545
1.94	2.54	2	2.512013	0.023987
2.10	2.51	2	2.55456	-0.04656
1.84	2.55	2	2.501675	0.044325
1.88	2.52	2	2.505003	0.013997
1.81	2.53	2	2.50083	0.03117
1.94	2.59	2	2.51309	0.07791
2.01	2.51	2	2.525656	-0.01766
1.87	2.59	2	2.50391	0.09009
1.97	2.59	2	2.517776	0.068224
2.06	2.48	2	2.540085	-0.06108
1.90	2.46	2	2.507308	-0.04731
1.92	2.47	2	2.50945	-0.04445
1.95	2.45	2	2.51372	-0.06072
1.87	2.46	2	2.503833	-0.03983
2.05	2.55	2	2.536979	0.015021
2.17	2.46	2	2.57933	-0.11833
2.14	2.50	2	2.568908	-0.07091
1.82	2.58	2	2.50088	0.07612
2.19	2.45	2	2.58625	-0.13325
1.95	2.57	2	2.513479	0.058521
1.85	2.49	2	2.50245	-0.01045
1.97	2.47	2	2.518006	-0.05001
2.04	2.56	2	2.534762	0.020238
2.03	2.54	2	2.53143	0.01257
1.98	2.58	2	2.520298	0.061702
1.94	2.56	2	2.513007	0.046993
1.87	2.57	2	2.503484	0.067516
1.78	2.49	2	2.500679	-0.00868
1.84	2.48	2	2.502029	-0.02503

1.88	2.56	2	2.504687	0.058313
1.78	2.57	2	2.500681	0.070319
1.78	2.60	2	2.500688	0.098312
1.95	2.51	2	2.514739	-0.00274
1.89	2.51	2	2.506168	-0.00017
1.88	2.57	2	2.504901	0.061099
2.10	2.50	2	2.554217	-0.05322
2.03	2.59	2	2.530873	0.059127
1.92	2.58	2	2.508907	0.070093
1.92	2.51	2	2.509116	0.004884
1.77	2.56	2	2.500932	0.062068
1.81	2.56	2	2.50076	0.06224
1.67	1.84	3	1.810211	0.029789
1.86	1.81	3	1.805067	0.001933
1.75	1.76	3	1.803225	-0.04122
1.80	1.86	3	1.802407	0.060593
1.83	1.73	3	1.803152	-0.07315
1.90	1.73	3	1.808215	-0.07522
1.81	1.88	3	1.802483	0.079517
1.87	1.87	3	1.805763	0.059237
1.76	1.89	3	1.803121	0.086879
1.82	1.85	3	1.802743	0.042257
1.78	1.82	3	1.802513	0.020487
1.91	1.78	3	1.809445	-0.02544
1.77	1.89	3	1.802793	0.084207
1.84	1.87	3	1.803723	0.069277
1.84	1.71	3	1.803749	-0.09475
1.75	1.71	3	1.803232	-0.09823
1.85	1.78	3	1.804398	-0.0294
1.80	1.86	3	1.802424	0.059576
1.68	1.90	3	1.809496	0.087504
1.76	1.73	3	1.80285	-0.07285
1.80	1.82	3	1.80245	0.01655
1.79	1.78	3	1.802395	-0.02639
1.87	1.89	3	1.805705	0.088295
1.80	1.87	3	1.802469	0.065531
1.83	1.87	3	1.803368	0.064632
1.83	1.79	3	1.803001	-0.009
1.75	1.78	3	1.803515	-0.02051
1.77	1.76	3	1.802632	-0.04763
1.78	1.71	3	1.802528	-0.09153
1.78	1.87	3	1.802508	0.070492
1.82	1.86	3	1.802959	0.060041

1.81	1.90	3	1.802533	0.097467
1.75	1.90	3	1.803303	0.095697
1.87	1.81	3	1.805746	0.005254
1.91	1.85	3	1.809682	0.044318
1.77	1.89	3	1.802781	0.086219
1.76	1.87	3	1.803071	0.066929
1.86	1.75	3	1.804736	-0.05574
1.75	1.79	3	1.803431	-0.01343
1.74	1.73	3	1.80399	-0.07799
1.72	1.89	3	1.80526	0.08574
1.76	1.82	3	1.802975	0.018025
1.81	1.75	3	1.802569	-0.05657
1.89	1.83	3	1.807429	0.026571
1.84	1.82	3	1.803485	0.020515
1.79	1.77	3	1.802425	-0.03043
1.83	1.72	3	1.803269	-0.08027
1.81	1.83	3	1.802479	0.031521
1.83	1.80	3	1.803083	0.000917
1.77	1.85	3	1.802595	0.051405
1.84	1.80	3	1.803553	0.000447
1.84	1.87	3	1.803619	0.066381
1.85	1.81	3	1.804046	0.005954
1.86	1.81	3	1.804721	0.007279
1.73	1.88	3	1.804329	0.070671
1.81	1.78	3	1.802515	-0.02151
1.79	1.73	3	1.802395	-0.0754
1.78	1.71	3	1.802443	-0.09644
1.81	1.85	3	1.802576	0.048424
1.80	1.82	3	1.802419	0.021581
1.86	1.84	3	1.804622	0.036378
1.71	1.74	3	1.805925	-0.06292
1.86	1.73	3	1.805037	-0.07804
1.81	1.70	3	1.802607	-0.09961
1.87	1.77	3	1.805769	-0.03577
1.76	1.82	3	1.802852	0.015148
1.79	1.78	3	1.802394	-0.02439
1.85	1.79	3	1.80436	-0.01736
1.76	1.88	3	1.802865	0.078135
1.77	1.77	3	1.802663	-0.03266
1.86	1.80	3	1.805143	-0.00214
1.71	1.86	3	1.805773	0.051227
1.79	1.78	3	1.802395	-0.0234
1.74	1.82	3	1.804139	0.019861

1.72	1.87	3	1.805054	0.066946
1.87	1.89	3	1.805485	0.084515
1.76	1.73	3	1.802903	-0.0739
1.80	1.89	3	1.802449	0.082551
1.80	1.80	3	1.802477	-0.00448
1.76	1.75	3	1.802825	-0.05083
1.80	1.79	3	1.802467	-0.01047
1.73	1.90	3	1.804562	0.091438
1.86	1.80	3	1.805143	-0.00614
1.74	1.77	3	1.803756	-0.03776
1.79	1.83	3	1.802397	0.024603
1.72	1.75	3	1.805209	-0.05721
1.81	1.72	3	1.802635	-0.08764
1.73	1.73	3	1.804649	-0.07865
1.91	1.73	3	1.810657	-0.08466
1.78	1.73	3	1.802432	-0.07243
1.80	1.73	3	1.802424	-0.07442
1.71	1.83	3	1.806542	0.021458
1.75	1.74	3	1.803585	-0.06758
1.77	1.77	3	1.802592	-0.03359
1.83	1.88	3	1.8031	0.0759
1.89	1.80	3	1.80803	-0.01303
1.76	1.83	3	1.80311	0.03089
1.81	1.73	3	1.802609	-0.06861
1.86	1.74	3	1.805236	-0.06724
1.89	1.71	3	1.807131	-0.09913
